# Comprehensive machine learning and experimental verification reveal the mechanism of action of autophagy-related genes FIZ1 and FBXO21 in acute kidney injury

**DOI:** 10.7717/peerj.20707

**Published:** 2026-02-02

**Authors:** Yunqi Bai, Lili Zhang, Bo Nie, Yixin Su, Jingwei Zhou

**Affiliations:** 1 Beijing University of Chinese Medicine and Pharmacology, Beijing, China; 2 First Affiliated Hospital, Beijing University of Chinese Medicine and Pharmacology, Beijing, China

**Keywords:** Acute kidney injury, GEO datase, Bioinformatics analysis, FIZ1, FBXO21, Autophagy

## Abstract

**Background:**

Acute kidney injury (AKI) is a serious disease with a high incidence and easy induction. The search for innovative biomarkers and treatment methods is of great significance for improving the prognosis of patients. Autophagy is closely related to the occurrence and development of AKI. This study aims to explore the role of autophagy-related genes (ARGs) as potential biomarkers and therapeutic targets in AKI.

**Methods:**

In this study, the gene microarray data of the GEO dataset were used to explore the molecular profile of AKI, and three machine learning algorithms were used to screen autophagy-related feature genes. To further validate the reliability of the screening results, we constructed a cisplatin-induced AKI rat model to validate potential biomarkers of machine learning screening.

**Results:**

Machine learning analysis identified 17 differentially expressed ARGs and selected the core genes FIZ1 and FBXO21, with area under curve (AUC) values both exceeding 0.7 (95% CI [0.706–0.899]). Immune analysis revealed that the number of Mast cells resting significantly decreased in AKI samples compared to normal samples (*P* < 0.05). Electron microscopy observations of the cisplatin-induced AKI rat model indicated thickening of the basement membrane, fusion of foot processes, and swelling and rupture of mitochondria in the model group, suggesting a correlation between AKI and mitochondrial autophagy; Western blot results indicated a significant increase in the expression of FIZ1 and a significant decrease in FBXO21 in the AKI group (*P* < 0.01). The results of IHC staining were also consistent with those of Western blot results.

**Conclusion:**

This study highlights the significant role of ARGs in AKI and identifies FIZ1 and FBXO21 as promising biomarkers with high diagnostic potential, offering new insights into the molecular mechanisms underlying AKI.

## Introduction

Acute kidney injury (AKI) is a common clinical syndrome affecting approximately 10–15% of hospitalized patients and 30–60% of critically ill patients, with a strong association to high mortality rates (Al-Jaghbeer et al., 2018; Hoste et al., 2018; Rossiter et al., 2024). The diagnostic criteria for AKI in the 2012 KDIGO guidelines are an increase of ≥ 26.5 µmol/L in serum creatinine within 48 h, or an increase of more than 1.5 times the baseline value within 7 days, or a urine output of less than 0.5 ml/(kg h) for six consecutive hours. Currently, AKI diagnosis primarily relies on monitoring urine output and serum creatinine (Scr) levels (Ugwuowo et al., 2020). These two indicators only show abnormality after kidney injury. The increase in Scr level is usually delayed, and urine volume monitoring also has problems of insufficient accuracy or being disturbed by multiple factors, and thus cannot reflect early AKI damage. As AKI is caused by various etiologies, including low cardiac output, sepsis, major surgery, drug toxicity, insufficient perfusion and inflammation. These factors vary greatly in severity and progression (Pickkers et al., 2021). These variables have led to the inability to accurately diagnose and effectively treat early AKI at an early stage. Despite extensive research, the pathophysiological mechanism of AKI remains incompletely understood, which hinders the development of early detection and effective drug treatments. Early detection of AKI in clinical practice is beneficial for the subsequent treatment plan and treatment outcome of patients (Al-Jaghbeer et al., 2018). Therefore, the development of biomarkers capable of early detection of AKI is particularly urgent, which is the key to breaking through the current predicament in the diagnosis and treatment of AKI.

Autophagy is a cellular lysosomal degradation pathway that facilitates the formation of autophagosomes and autolysosomes to degrade cytoplasmic components, thus maintaining cellular homeostasis (Mizushima, 2018). Dysregulation of autophagy has been observed in both animal and clinical studies of AKI and chronic kidney disease (CKD) (Choi, 2020; Tang et al., 2020). Research on cisplatin-induced AKI animal models has shown that autophagy occurs at the early stage of cisplatin-induced AKI animal models, playing a crucial role in the prevention and treatment of kidney damage. Knockout of autophagy related genes, such as Atg7 or Atg5, can exacerbate kidney damage in cisplatin-induced AKI models, while administration of autophagy inhibitors can ameliorate renal injury (Ji et al., 2015; Yu et al., 2023a). Moreover, chloroquine alleviates renal tubular damage and renal function decline in rats with cisplatin-induced AKI animal model by activating autophagy (Herzog et al., 2012; Jiang et al., 2012), further supporting the protective role of autophagy in kidney health. Increasing evidence suggests a close link between autophagy and kidney injury in AKI (Yuan et al., 2024), with various compounds showing potential in mitigating AKI by modulating autophagy (Shao et al., 2023). This study aims to identify autophagy-related genes (ARGs) that affect the development of AKI through bioinformatics methods, and validate the selected biomarkers based on cisplatin-induced AKI animal models, providing specific diagnostic markers for clinical AKI and offering a theoretical basis for discovering new pharmacodynamic targets for drug development.

In this study, we analyzed mRNA microarray data from the GEO dataset to delineate the molecular characteristics of AKI. Using three machine learning algorithms (Least Absolute Shrinkage and Selection Operator (LASSO), Random Forest, support vector machine-recursive feature elimination (SVM-RFE)), we identified AKI autophagy-related features (FIZ1, FBXO21). We constructed an *in vitro* cisplatin-induced AKI rat injury model to experimentally validate potential biomarkers. This experiment preliminarily confirmed the accuracy of the genes screened by machine learning, FIZ1 and FBXO21. This result is helpful for improving our understanding and diagnostic ability of AKI research.

## Methods

### Retrieval of AKI-related microarray datasets

In this study, we retrieved transcriptomic datasets related to AKI published in the past decade from the Gene Expression Omnibus (GEO) database (https://www.ncbi.nlm.nih.gov/). The selected datasets include GSE61739, GSE139061, and GSE53769. GSE61739 contains 24 normal control samples and 24 AKI biopsy samples, utilizing the GPL20301 platform. GSE53769 includes 10 normal control samples and eight AKI biopsy samples, utilizing the GPL16686 platform. GSE139061 comprises nine normal control samples and 39 AKI biopsy samples, also using the GPL20301 platform. GSE139061 and GSE61739 were designated as discovery cohorts, while GSE53769 served as the validation cohort.

### Removal of batch effects

To mitigate batch effects arising from study design, sequencing platforms, and technical replicates, we merged the GSE139061 and GSE61739 datasets into a combined dataset consisting of 63 AKI patients and 33 normal control samples. Batch effect correction was performed using the “sva” package (version 3.52.0) in R (version 4.3.1; R Core Team, 2023) (Leek et al., 2012), and the correction effect was assessed through visualization using the “ggplot2” and “limma” package (version 3.5.1 and 3.60.6). DEGs in GSE139061 and GSE61739 were further analyzed for upregulation and downregulation using the “RobustRankAggreg” and “pheatmap” tools.

### Identification of autophagy-related differentially expressed genes

Differential gene expression analysis was conducted using the “limma” package (version 3.60.6) in R to compare the AKI and normal groups within the merged GSE139061 and GSE61739 datasets, identifying DEGs. The filtering criteria were set to —logFC— > 1 and *p* < 0.05. Genes with logFC > 1 and *p* < 0.05 were classified as upregulated DEGs, while those with logFC < −1 and *p* < 0.05 were categorized as downregulated DEGs. Based on these criteria, we identified the DEGs from the combined dataset. Subsequently, we performed an intersection analysis between the DEGs and ARGs to identify autophagy-related DEGs. ARGs were sourced from the Human Autophagy Database (HADb, https://www.autophagy.lu/) (Safran et al., 2010) and GeneCards. The HADb database provides 232 ARGs (Table S1), while GeneCards was queried with the keyword “Autophagy” and filtered for protein-coding genes with a score greater than 1, yielding 4,154 ARGs. After merging the two databases, we obtained a total of 4,185 ARGs (Table S2). The results of differential expression analysis were visualized using R packages “ggplot2” “pheatmap” and “RCircos” (version 3.5.1, 1.0.12, and 1.2.2) including volcano plots, heatmaps, and chromosome distribution maps.

### Identification and validation of AKI-related biomarkers

After identifying the ARGs-AKI-related DEGs, we proceeded to identify the most relevant biomarkers for AKI. We applied three machine learning algorithms—LASSO, Random Forest (RF), and Support Vector Machine (SVM)—to screen for biomarkers capable of effectively distinguishing AKI from normal samples. The parameters for these algorithms were set according to the guidelines provided by their respective R packages. Variable selection for ARGs-AKI-related DEGs was performed using the “glmnet” “caret” and “RandomForest” packages (version 4.1-8, 6.0-94, and 4.7-1.2) in R, with parameters set as recommended in the corresponding R package documentation. The LASSO algorithm is performed using “glmnet” packages, combined with ten-fold cross-validation to identify important genes. For the RF algorithm, we used the “RandomForest” package and selected the top 10 genes as our main candidate genes. SVM-RFE algorithm was used in e1071 package (version 1.7-16) to determine the optimal gene subset according to the accuracy. Additionally, receiver operating characteristic (ROC) curve analysis was conducted using the “pROC” (version 1.18.5) package in R to evaluate the diagnostic accuracy of the biomarkers identified in both the training and test cohorts. The algorithm with the highest accuracy was chosen for further analysis in the test cohort. The GSE53769 dataset was used as the validation cohort to assess the specificity and sensitivity of the biomarkers identified in the training cohort.

### Construction of nomogram and risk prediction model

To evaluate the contribution of key DEGs to the diagnosis of AKI, this study performed logistic regression analysis on the key DEGs from the combined dataset and constructed a logistic regression model. Based on the results of the logistic regression analysis, a nomogram was constructed using the “rms” (version 1.18.5) package in R. The performance of the nomogram was assessed through calibration curves, and DCA was conducted to evaluate the accuracy and clinical utility of the model.

### Gene set enrichment analysis

Gene set enrichment analysis (GSEA) was conducted using the “GSEA” package (version 1.66.0) in R to investigate the pathways associated with the core genes. The correlation between these core genes and other genes in the GSE53769 dataset was calculated, and all genes were ranked by correlation in descending order to form the gene set for analysis. KEGG signaling pathway sets were used as predefined gene sets, as they provide comprehensive and well-annotated biological pathway resources. This approach helps evaluate the enrichment of identified genes in known biological pathways and further elucidates their functional roles.

### Gene set variation analysis

Gene set variation analysis (GSVA) is one of the GSEA algorithms that explores differences in biological pathways between different clusters of samples based on enrichment scores. The “GSVA” R package (version 1.52.3) was used to perform functional enrichment analysis on AKI disease samples from the combined dataset to identify enriched pathways. The “c2.cp.kegg.v7.4.symbols.gmt” file from the MsigDB database was used for this analysis. A corrected *P* value < 0.05 was considered statistically significant, indicating differences between clusters.

### Immune infiltration analysis

The “CIBERSORT” tool (version 1.03) in R was used to predict the proportions of 22 different types of immune cells infiltrating each tissue sample in the dataset. For each sample, the proportions of all evaluated immune cell types were normalized to sum to 1, ensuring a comprehensive assessment of immune cell infiltration.

### Establishment of AKI animal model and validation of core genes

Twelve male SPF-grade SD rats, aged 6 weeks, weighing 180 to 200 grams, were purchased from Beijing Vital River Laboratory Animal Technology Co., Ltd. (SCXK [Beijing] 2021-0006). The rats were housed in facilities at Beijing University of Chinese Medicine under conditions of good ventilation, with a 12-hour light/dark cycle, and constant humidity and temperature (22–25 °C). The density of rats was moderate, with four rats per cage. The experimental protocol was approved by the Animal Ethics Committee of Beijing University of Chinese Medicine (BUCM-2024022003-1196), and all experiments followed national and international guidelines for animal care. After one week of acclimatization, rats were intraperitoneally injected with cisplatin (seven mg/kg) or 0.9% saline (Qu et al., 2019). General health status was monitored post-injection, and after 72 h, the rats were anesthetized with 1% tribromoethanol. After the rats had no pedal reflex, blood was drawn from the abdominal aorta and they were sacrificed. The rats were killed by rapid blood loss. Blood samples were collected *via* the abdominal aorta for Scr and BUN testing. The connective tissue was removed, and both kidneys were weighed and photographed. The right kidney was preserved in 4% paraformaldehyde for pathological examination, while the left kidney index was calculated as the left kidney wet weight-to-body weight ratio. Molecular biology experiments were also performed.

For protein extraction, 30 mg of kidney tissue was homogenized on ice and lysed using RIPA buffer for 30 min, followed by centrifugation at 15,000×g for 20 min at 4 °C. The supernatant was collected for protein quantification using a BCA protein assay kit. Equal amounts of total protein were denatured at 100 °C for 10 min and separated by SDS-PAGE. Proteins were transferred to a nitrocellulose membrane, blocked overnight with 10% skim milk at 4 °C, and incubated with primary antibodies (FIZ1; 15826-1-AP, diluted 1:1,000; Proteintech), FBXO21 (abcam, ab179818, diluted 1:10000)) at room temperature. After incubation with ECL substrate for 1 min, the chemiluminescent signals were detected. ImageQuant™ LAS 4000 (Cytiva, Tokyo, Japan) was used for imaging, and ImageJ and Photoshop were utilized for semi-quantitative analysis of the protein blots.

Fresh kidney cortex tissue (one mm^3^) was fixed in 4% glutaraldehyde at 4 °C for 24 h, then rinsed three times with 0.1 M phosphate buffer (pH 7.4) for 15 min each. The tissue was fixed in 1% osmium tetroxide in the dark at room temperature for 2 h, followed by rinsing with phosphate buffer and dehydration in a graded ethanol series. After embedding in acetone and embedding media, the tissue was stained with toluidine blue and observed under a light microscope. Ultrathin sections (60–80 nm) were prepared and stained with 2% uranyl acetate in absolute ethanol for 8 min, followed by staining with 2.6% lead citrate. Imaging was performed using a HT7800/HT7700 transmission electron microscope (Hitachi, Tokyo, Japan) to observe ultrastructural details such as podocyte foot processes, mitochondria, lysosomes, lipid droplets, rough endoplasmic reticulum, and MAM structures.

The right kidney of the rats was fixed in 4% paraformaldehyde for 24 h, rinsed with tap water for 30 min, dehydrated in graded alcohol, and embedded in paraffin. The tissue was sectioned at four µm and stained with H&E and immunohistochemistry using the following antibodies: FIZ1 (15826-1-AP, diluted 1:100; Proteintech) and FBXO21 (Abcam, ab179818, diluted 1:50) according to the kit instructions. Pathological morphology of the kidney was examined under a light microscope, and images were captured at 400 × magnification from six random fields of view.

### Statistical analysis

All data processing and analysis in this study were performed using R software (R Core Team, 2023). For comparison of continuous variables between two groups, independent Student’s t-tests were used for normally distributed variables, and the Wilcoxon rank-sum test was used for non-normally distributed variables. All statistical tests were two-sided, and a *P* value < 0.05 was considered statistically significant.

## Results

### Identification and enrichment analysis of DEGs

**Figure 1:**
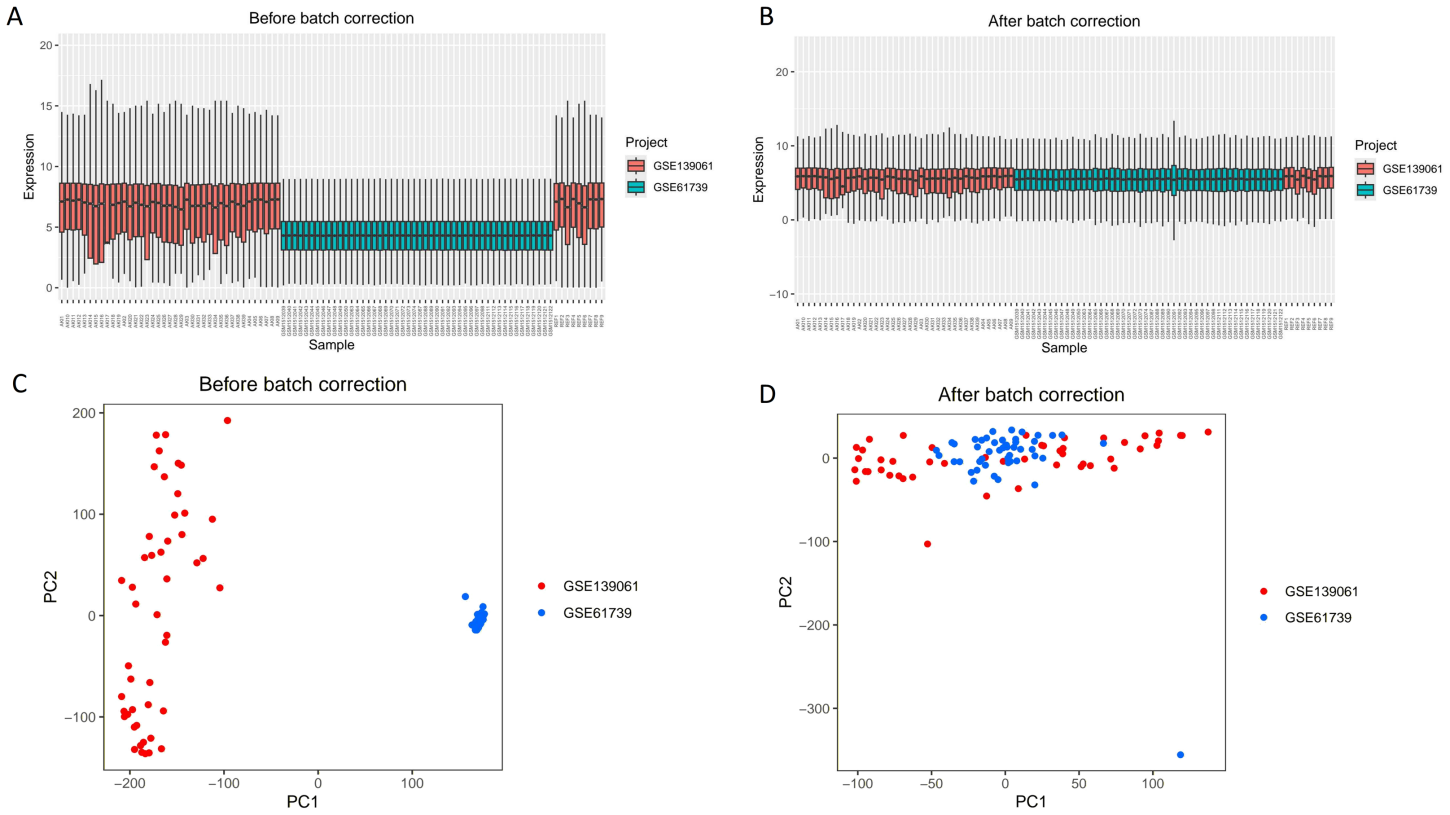
Boxplots and PCA results of the GSE139061 and GSE61739 combined datasets before and after batch effect removal. (A) Boxplot of GSE139061 and GSE61739 Combined dataset before batch effect removal. Without batch effect correction, there is a clear distribution difference between the GSE139061 samples (shown in red) and the GSE61739 samples (shown in blue). (B) Boxplot of GSE139061 and GSE61739 combined dataset after batch effect removal. Following batch effect correction, the distribution differences between the red and blue samples significantly decrease, indicating that batch effects have been effectively mitigated. (C) PCA of GSE139061 and GSE61739 combined dataset before batch effect removal. PCA reveals that the red and blue samples are clearly separated along the principal component axes, likely due to batch effects causing sample differences. (D) PCA of GSE139061 and GSE61739 combined dataset after batch effect removal. After batch effect correction, the PCA plot shows that the red and blue samples are more closely grouped, suggesting that batch effects have been minimized, allowing for a better representation of the natural variability between samples. Red indicates GSE139061 samples, and blue indicates GSE61739 samples.

As shown in Fig. 1, this study first merged the GSE139061 and GSE61739 datasets to construct an integrated GEO dataset. To eliminate batch effects, we used the “SVA” package in R (Figs. 1A, 1B). After batch effect correction, the effectiveness of the processing was validated by principal component analysis (PCA) analysis (Figs. 1C, 1D). These results show that by using the “SVA” package in R for batch effect correction, we effectively reduced technical variation between the different datasets, providing a more accurate data foundation for subsequent analysis. The differentiation between red and blue samples represents the samples from the GSE139061 and GSE61739 datasets, respectively, and the color coding helps intuitively compare the differences before and after the correction.

**Figure 2:**
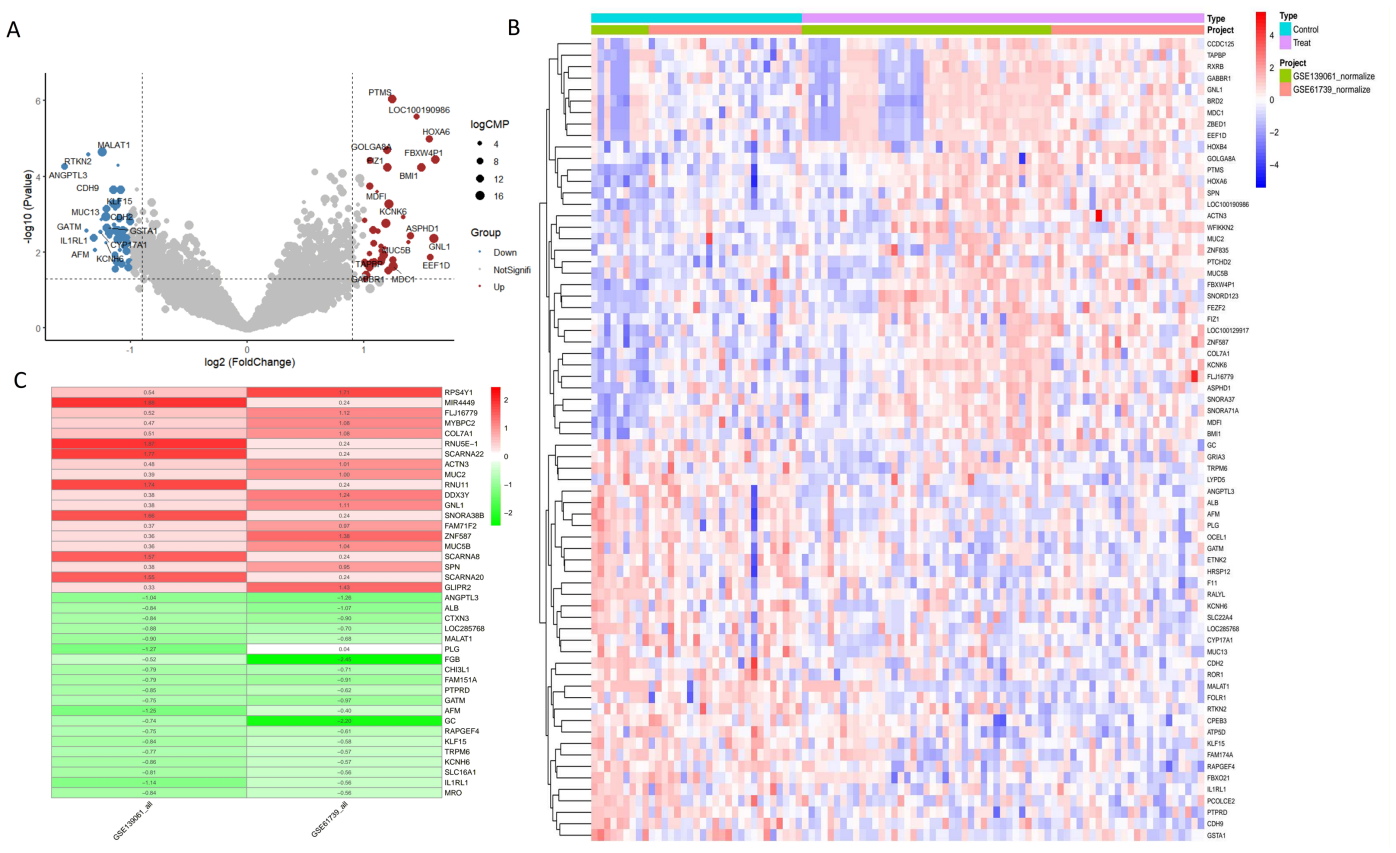
DEGs analysis of GSE53769 and GSE139061 combined datasets. (A) Volcano plot of DEGs in GSE53769 and GSE139061 combined datasets. The volcano plot shows the significance and fold changes of gene expression variations between the two datasets. (B) Heatmap of DEGs in GSE53769 and GSE139061 combined datasets. This heatmap visually presents the differential expression patterns of genes across both datasets. (C) Heatmap of DEGs using the RRA ranking method in GSE53769 and GSE139061 combined datasets. This heatmap further validates the expression differences of DEGs using the Relative Ranking Analysis (RRA) method.

**Figure 3:**
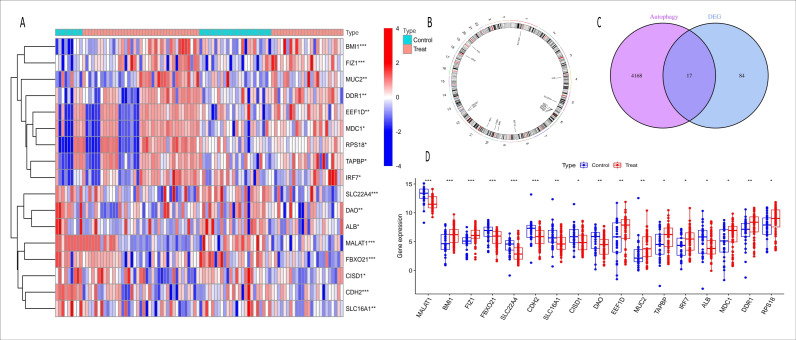
Heatmap, chromosome map, venn diagram, and boxplot of ARGs-AKI related DEGs. (A) Heatmap of ARGs-AKI related DEGs. The heatmap shows the expression patterns of autophagy-related DEGs in AKI samples. The color gradient represents relative gene expression levels, highlighting the ARGs with significant differential expression in AKI. (B) Chromosome map of ARGs-AKI related DEGs. This map shows the physical locations of the ARGs-AKI related DEGs on human chromosomes, providing insight into their genetic background and potential genomic impacts. (C) Venn diagram of DEGs and ARGs in the GSE53769 and GSE139061 combined datasets. The venn diagram illustrates the intersection between DEGs and ARGs, identifying the ARGs-DEGs that may play a crucial role in AKI and serve as potential targets for future research. (D) Boxplot of ARGs-DEGs Intersection. The boxplot displays the statistical significance (*p* < 0.0001) of the expression changes of these genes, confirming their significant involvement in AKI.

In the combined analysis of the GSE139061 and GSE61739 datasets, 46 upregulated and 55 downregulated DEGs were identified (Figs. 2A, 2B, ARGs-DEGs C, Table S3). Through the intersection analysis of ARGs and DEGs, 17 ARGs-DEGs were further selected (Figs. 3A–3C, Table S4). The distribution of these across human chromosomes is shown in the chromosome map (Fig. 3B). All ARGs-DEGs displayed significant differences between the two groups (*P* < 0.001), as shown in Fig. 3D. These analysis results not only reveal key gene expression changes in AKI but also provide important clues for subsequent functional studies and the discovery of potential therapeutic targets through intersection analysis with autophagy related genes.

### Machine learning analysis to identify core genes

**Figure 4:**
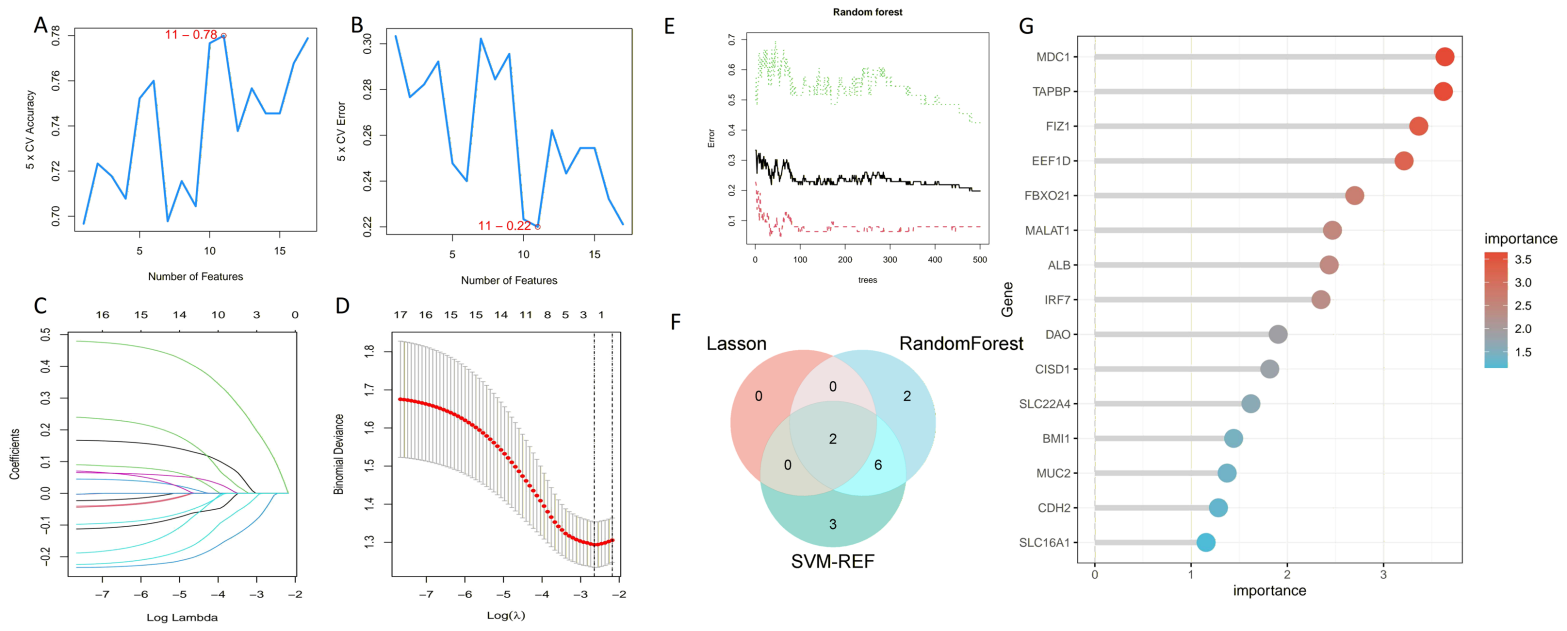
Machine learning algorithms for selecting key ARGs-DEGs. (A, B) SVM-RFE algorithm to select key ARGs-DEGs. (C, D) LASSO algorithm for key ARGs-DEGs selection. (E) Confidence interval of random forest error rate. (G) Importance ranking of genes in random forest. (F) Intersection of LASSO, SVM, and random forest algorithms. This diagram shows the overlap of key ARGs-DEGs identified by the LASSO, SVM, and random forest algorithms.

**Figure 5:**
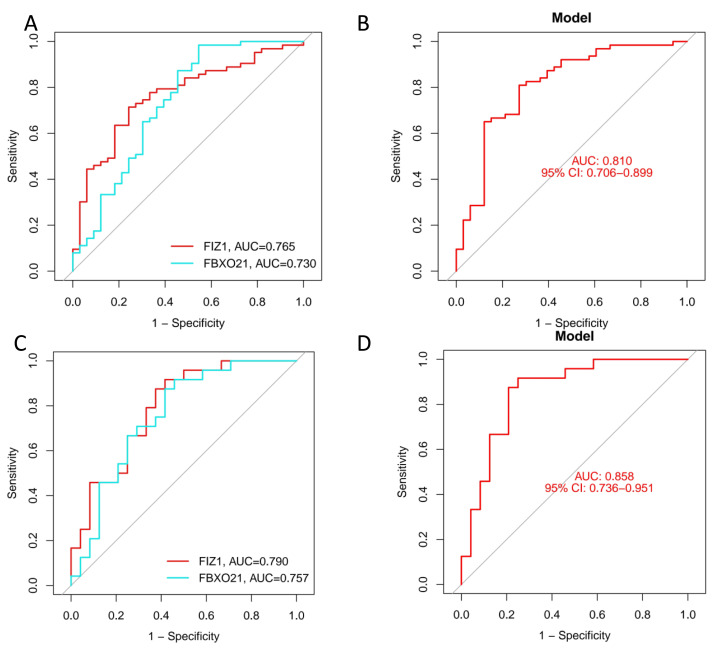
ROC curves of two genes in the training and validation cohorts. (A–B) ROC curves of two genes in the training cohort. (C–D) ROC curves of two genes in the validation cohort. These ROC curves demonstrate the diagnostic performance of the two identified genes in both the training and validation cohorts.

We employed three machine learning algorithms—LASSO, SVM-RFE, and Random Forest—to predict the DEGs most related to autophagy and AKI. The SVM-RFE analysis identified 11 genes (Figs. 4A, 4B), followed by two genes from LASSO regression analysis (Figs. 4C, 4D), and 17 genes from the Random Forest model (Figs. 4E, 4G). Since each feature selection method has its own advantages and limitations, we performed an intersection analysis of the genes selected by all three methods to identify candidate genes related to ARGs-AKI. By cross-referencing the important genes identified by the three models, we identified two biomarkers: FIZ1 and FBXO21 (Fig. 4F). The receiver operating characteristic (ROC) curve of FIZ1 and FBXO21 in the training cohort showed area under curve (AUC) values greater than 0.7 (95% CI [0.706–0.899]), indicating high diagnostic potential (Figs. 5A–5B). The GSE53769 dataset was used as a validation cohort, and the ROC curves for FIZ1 and FBXO21 in the validation cohort also showed AUC values greater than 0.7 (95% CI [0.736–0.951]), further confirming their high diagnostic accuracy (Figs. 5C–5D).

### Establishment and evaluation of a nomogram model for diagnosing key genes and assessing efficacy

**Figure 6:**
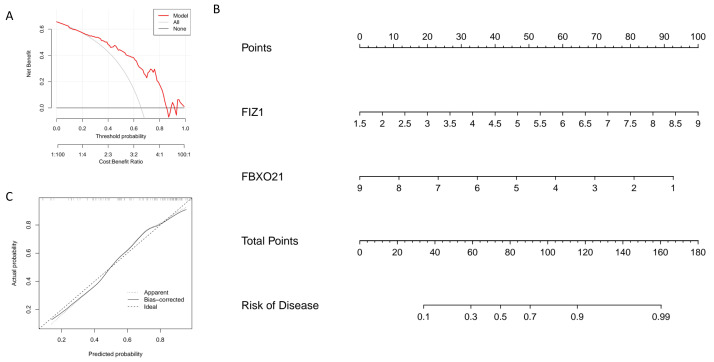
Establishment and evaluation of the nomogram model for diagnosing key genes FIZ1 and FBXO21. (A) DCA: Evaluates the clinical applicability of the nomogram model. (B) Nomogram based on FIZ1 and FBXO21: Displays the prediction of individual AKI risk based on the expression levels of FIZ1 and FBXO21. (C) Calibration curve: Compares the predicted probabilities from the nomogram model with the actual occurrence probabilities, assessing the model’s accuracy.

In this study, we constructed a nomogram model based on two key genes, FIZ1 and FBXO21, to predict the risk of autophagy-related AKI. In the nomogram, the expression value of each significant gene corresponds to a specific score, and the total score is calculated by summing the scores of all feature genes. This cumulative score reflects an individual’s risk of developing autophagy-related AKI (Fig. 6B). The calibration curve demonstrates that the predicted probabilities from the nomogram are highly consistent with the ideal model (Fig. 6C), indicating the model’s high accuracy. Decision curve analysis (DCA) further supports the utility of the nomogram, suggesting it offers an advantage for diagnosing autophagy-related AKI (Fig. 6A). Based on these findings, we conclude that FIZ1 and FBXO21 exhibit excellent diagnostic performance for predicting autophagy-related AKI.

### Immune cell infiltration analysis

**Figure 7:**
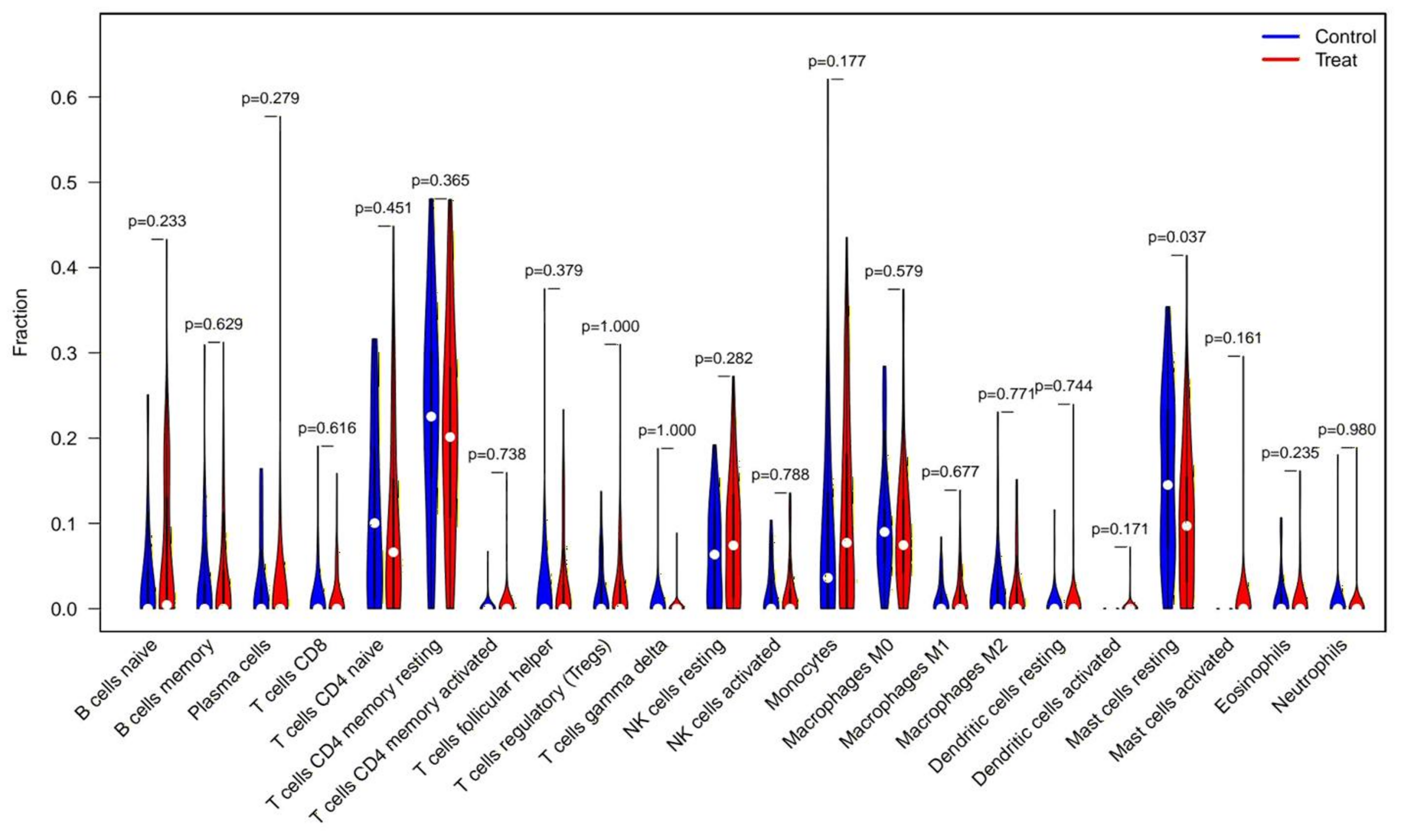
CIBERSORT analysis of immune cell type distribution in AKI samples and the association between differentially infiltrated immune cells and the expression of FIZ1 and FBXO21. CIBERSORT analysis of 22 immune cell types: this graph illustrates the relative proportions of various immune cell types in AKI samples, offering a comprehensive view of the immune microenvironment in AKI. By comparing AKI samples with normal samples, significant alterations in immune cell populations are identified.

CIBERSORT analysis revealed significant differences in the immune microenvironment between AKI and normal samples (Fig. 7). Specifically, the proportion of resting mast cells was significantly reduced in AKI samples compared to normal samples.

### Specific signaling pathways associated with FIZ1 and FBXO21

**Figure 8:**
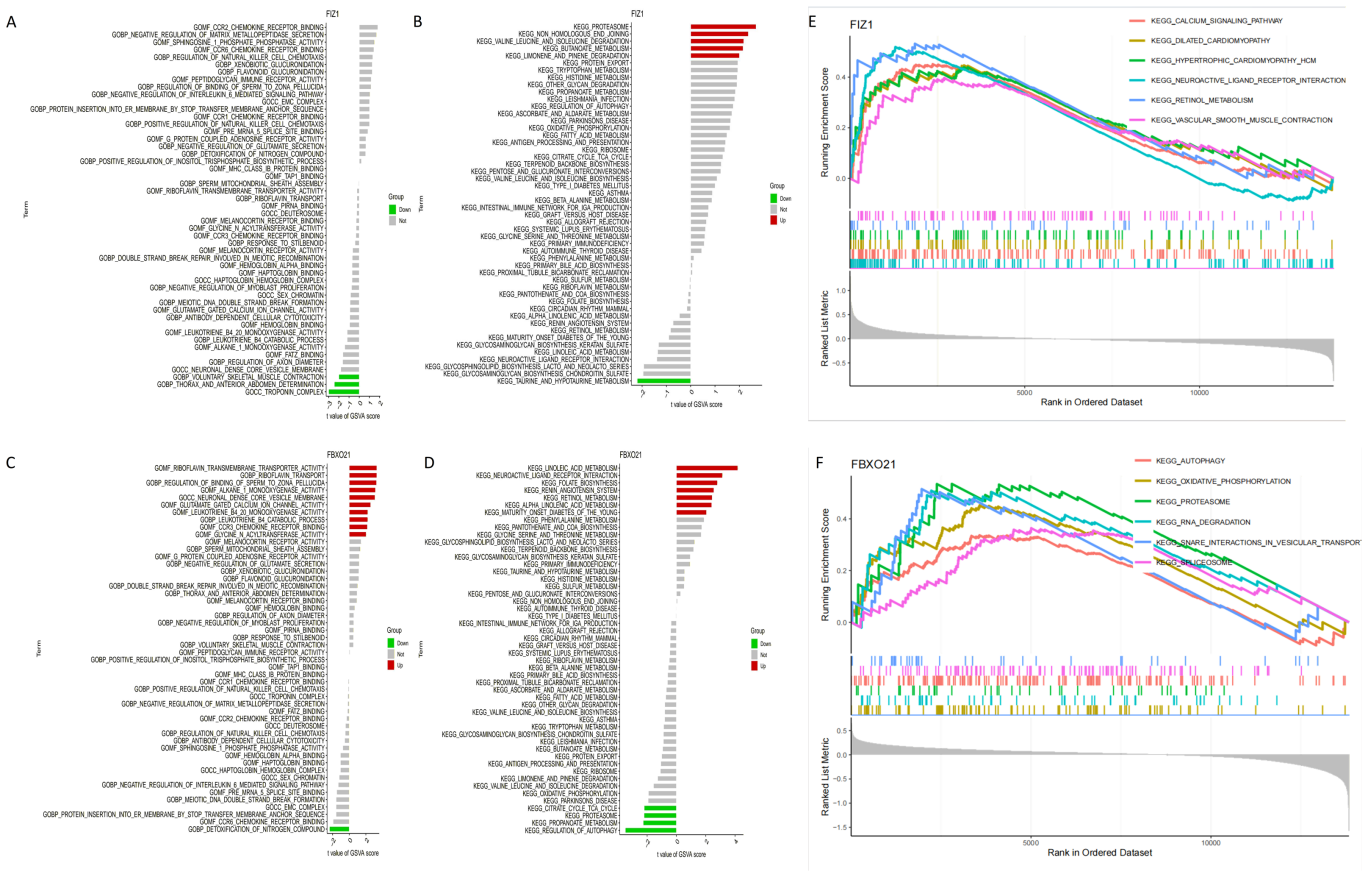
Analysis of signaling pathways associated with FIZ1 and FBXO21. (A) Signaling pathways associated with high expression of FIZ1. (B) Signaling pathways associated with low expression of FIZ1. (C) Signaling pathways associated with high expression of FBXO21. (D) Signaling pathways associated with low expression of FBXO21. (E) The top five signaling pathways associated with high expression of FIZ1. (F) The top five signaling pathways associated with high expression of FBXO21.

The results of GSVA identified key signaling pathways associated with the expression levels of FIZ1 and FBXO21, offering insights into their roles in AKI. High expression levels of FIZ1 were primarily associated with the proteasome, tryptophan metabolism, protein export, and limonene and pinene degradation pathways, while low expression of FIZ1 correlated with taurine and hypotaurine metabolism pathways. High expression levels of FBXO21 were linked to linoleic acid metabolism, maturity onset diabetes of the young (MODY), alpha-linolenic acid metabolism, retinol metabolism, renin-angiotensin system, folate biosynthesis, and neuroactive ligand–receptor interaction pathways, whereas low FBXO21 expression was associated with regulation of autophagy, propanoate metabolism, proteasome, and citrate cycle (TCA cycle) pathways (Figs. 8A–8D).

GSEA further highlighted the top pathways associated with these genes: for FIZ1, the leading pathways included calcium signaling, dilated cardiomyopathy, hematopoietic cell lineage, neuroactive ligand–receptor interaction, oxidative phosphorylation, and proteasome, and for FBXO21, the pathways included autophagy, oxidative phosphorylation, proteasome, RNA degradation, snare interactions in vesiular transport,splceosome (Figs. 8E, 8F).

These findings suggest that FIZ1 and FBXO21 contribute to the pathogenesis of AKI through their involvement in critical biological processes, including calcium signaling, a pathway vital for intracellular communication and inflammatory responses, playing a pivotal role in kidney function and injury; oxidative phosphorylation, essential for energy metabolism, reflecting the mitochondrial function critical in renal tubular cells; and neuroactive ligand–receptor interaction, highlighting their roles in signal transduction and potential influence on inflammation and stress responses. The strong associations with these pathways underline the importance of FIZ1 and FBXO21 in inflammatory responses, energy metabolism, and cellular signaling, providing a theoretical foundation for exploring novel therapeutic strategies targeting these genes and advancing the understanding of molecular mechanisms underlying AKI.

### Validation of FIZ1 and FBXO21 expression in AKI rat models

**Figure 9:**
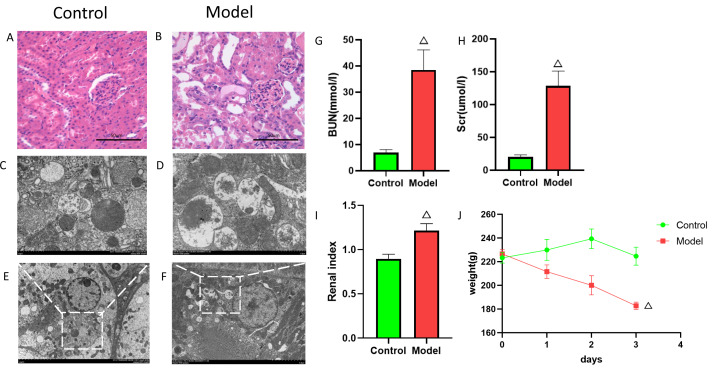
Establishment of cisplatin induced AKI model. (A, B) HE staining images of kidneys from cisplatin-induced AKI rat model. (C–F) Electron microscopy images of kidneys from cisplatin-induced AKI rat model. (G) Serum BUN levels (mean ± SD, *n* = 6). (H) Serum Scr levels (mean ± SD, *n* = 6); (I) Renal index (mean ± SD, *n* = 6). (J) Rat body weight changes (mean ± SD, *n* = 6).

To elucidate the mechanisms of FIZ1 and FBXO21 in AKI, we established a cisplatin-induced AKI rat model. After model establishment, the model group rats showed a significant decrease in body weight compared to the normal group (Fig. 9J), enlargement of the kidneys, and increased renal weight (Fig. 9I) (*p* < 0.05), with serum biochemistry indicating a significant increase in Scr and BUN (Figs. 9G, 9H). H&E staining revealed disarray in the arrangement of renal tubules, dilation of renal tubules, and fatty degeneration in the kidneys of the model group rats (Figs. 9A, 9B). Electron microscopy showed swelling of mitochondria, dissolution and fragmentation of cristae, and a decrease in matrix granules in the model group; the rough endoplasmic reticulum expanded in vesicular structures, and secondary lysosomes increased significantly; lipid droplets and autophagy were observed in the cytoplasm (Figs. 9C–9F). This evidence suggests that the AKI model is established and indicates a close relationship between AKI and autophagy.

**Figure 10:**
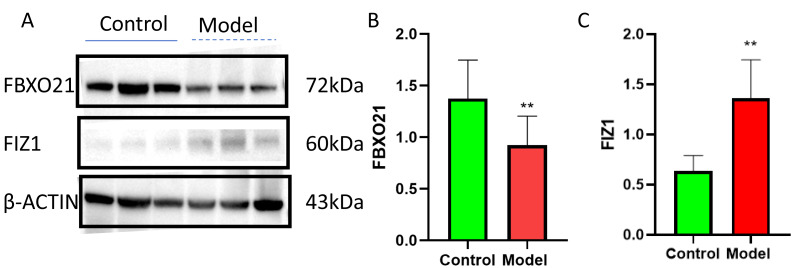
FBXO21 was underexpressed and FIZ1 was overexpressed in AKI. (A) Representative western blot images of FBXO21 and FIZ1 protein expression in kidney tissue. (B) Western blot analysis showing the protein expression levels of FBXO21 in kidney tissue (*n* = 6). (C) Western blot analysis showing the protein expression levels of FIZ1 in kidney tissue (*n* = 6). Data are presented as mean ± SD (*n* = 6). ** *P* < 0.01, compared with the normal group. These results demonstrate the altered expression of FBXO21 and FIZ1 in AKI, indicating their potential roles in the disease.

**Figure 11:**
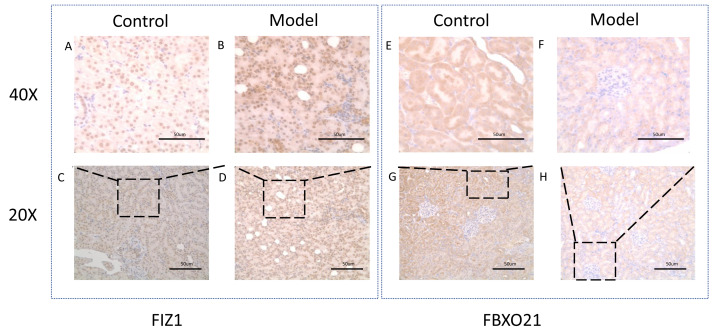
Representative image of FIZ1 and FBXO21 immunohistochemistry in cisplatin induced-AKI rat. (A, B, E, F) The model group was stained with IHC under 40X microscope. (C, D, E, F) The normal group was stained with IHC under 20X microscope.

Western blot (WB) detection revealed a significant decrease in the expression of FBXO21 (*p* < 0.05) (Figs. 10A, 10B) and a significant increase in the expression of FIZ1 (*p* < 0.05) (Figs. 10A, 10C) in the renal tissue of the AKI group. In addition, the IHC results also prove the above conclusions (Fig. 11). It consistent with our bioinformatics analysis results.

## Discussion

AKI is a common and serious condition among hospitalized patients that significantly increases the risk of developing CKD and end-stage renal disease (ESRD). Despite its prevalence, AKI remains a heterogeneous syndrome, and no specific, effective treatments are currently available. Therefore, early identification and diagnosis are critical to preventing the progression of AKI (Khan, Loi & Rosner, 2017). Recent research has highlighted a strong link between autophagy and AKI (Livingston et al., 2023; Su et al., 2023), with targeting autophagy showing potential for mitigating kidney damage (Liu et al., 2023; Deng et al., 2024; Zuo et al., 2024). Additionally, both autophagy and immune-related pathways have been found to play vital roles in AKI progression, suggesting that identifying novel immune-related biomarkers could contribute to the development of new therapeutic strategies (Pickles, Vigié & Youle, 2018; Choi, 2020; Livingston et al., 2023). Consequently, it is essential to explore new immune-related features for both diagnosing and treating AKI.

In this study, we utilized transcriptomic data from the GSE61739 and GSE139061 datasets to identify 101 DEGs, including 46 upregulated and 55 downregulated DEGs. By intersecting these DEGs with ARGs, we identified 17 autophagy-related DEGs (ARGs-DEGs). In this study, we employed three classic predictive models: LASSO, SVM, and RF. These methods have not only been widely validated in the medical field but also maintain robustness under small sample conditions, while balancing model performance, interpretability, and computational efficiency (Huang et al., 2025). Using machine learning algorithms, including LASSO, SVM, and RF, we identified two key genes, FIZ1 and FBXO21, which were further validated using the GSE53769 dataset. The area under the curve (AUC) values in the validation cohort were all greater than 0.7 (95% CI [0.706–0.899]), indicating that these two genes exhibit high diagnostic accuracy. We then constructed a nomogram to predict the risk of AKI based on the expression levels of FIZ1 and FBXO21. The calibration curve demonstrated that the nomogram model accurately predicted the risk of AKI based on these two genes. However, this model has certain limitations. Due to data constraints, validation was conducted using a single dataset, whereas multi-cohort external validation remains the gold standard for assessing model generalizability (Balachandran et al., 2015). Furthermore, the clinical translation of the model presents several challenges. Variability in patient-specific gene expression, differences in sample processing and detection methodologies, and the inherent complexity of clinical practice pose significant hurdles to fully integrating these factors into the nomogram (Zheng et al., 2024). In particular, AKI patients frequently present with multiple comorbidities and receive various pharmacological treatments. Drug interactions, along with the physiological impact of complications, may further influence the signaling pathways involving FIZ1 and FBXO21, adding complexity to disease progression and making precise outcome prediction more challenging. Furthermore, GSVA and GSEA revealed that FIZ1 and FBXO21 are involved in crucial pathways such as autophagy regulation, calcium signaling, and oxidative phosphorylation, all of which play significant roles in kidney injury diseases.

Autophagy is a vital cellular process that facilitates the clearance of damaged proteins and organelles, contributing to the maintenance of cellular homeostasis (Yorimitsu & Klionsky, 2005). As a degradation pathway, autophagy balances the biosynthesis and catabolism of macromolecules, protecting the body from various diseases (Li et al., 2022a). Mitochondrial autophagy, a specialized form of autophagy, targets damaged mitochondria and transports them to lysosomes for degradation (Pickles, Vigié & Youle, 2018). Kidney homeostasis, which requires substantial amounts of ATP produced primarily by mitochondria, depends heavily on renal tubular cells that contain a high density of mitochondria. These cells play a critical role in maintaining kidney function through mitochondrial autophagy (Bhargava & Schnellmann, 2017). Mitochondria are also involved in regulating second messengers, including calcium ions (Ca^2+^), cyclic adenosine monophosphate (cAMP), and reactive oxygen species (ROS) (Berridge, Lipp & Bootman, 2000; Gottlieb & Bernstein, 2016). Calcium homeostasis is particularly important in kidney function and the progression of kidney diseases, including AKI and CKD, ischemia/reperfusion (I/R) injury, autosomal dominant polycystic kidney disease (ADPKD), podocyte disease, and diabetic nephropathy (Ward, Brown & Valentovic, 2019; Hou et al., 2021; Ning et al., 2021; Liu et al., 2024; Ogasawara-Nosoko et al., 2024). Chen et al. (2025) confirmed that calcium signaling ameliorates acute kidney injury (AKI) through the regulation of the Ca^2^^+^-dependent calpain/HIF-1α/Notch pathway. Similarly, a study by Apizi et al., (2025) demonstrated that the hematopoietic cell lineage is closely associated with the severity of septic renal injury. Furthermore, Li et al. (2022b) established that neuroactive ligand–receptor interactions significantly influence renal function and play a critical role in the early stages of kidney injury. Additionally, various cellular processes, including oxidative phosphorylation, autophagy, proteasome activity, RNA degradation, SNARE interactions in vesicular transport, and the spliceosome, have been implicated as key mechanistic contributors to AKI (Tang et al., 2020). Our GSVA and GSEA analyses revealed that FIZ1 and FBXO21 are significantly enriched in pathways related to autophagy and calcium signaling, both of which are essential for maintaining kidney function.

FBXO21 is a member of the F-box protein family belonging to the FBXO sub-family and is one of the E3 ubiquitin ligases (Tekcham et al., 2020). E3 ubiquitin ligases are crucial components of the SKP1-CUL1-FBOX (SCF) complex, which mediates the ubiquitination cascade, a fundamental molecular process that underlies many biological mechanisms (Cardozo & Pagano, 2004). Previous studies have shown that another F-box protein, FBXW7α, is closely related to AKI (Mo et al., 2019). Shaw et al. (2020) suggested that FBXO21 could serve as a biomarker for AKI after kidney transplantation. This finding is of great significance for clinical determination of whether AKI occurs after kidney transplantation. At the molecular mechanism level, there is a complex relationship between FBXO21 and AKI. Additionally, studies by Yu et al. (2016) revealed that FBXO21 activates apoptosis signal-regulating kinase 1 (ASK1), which triggers the JNK and p38 pathways and plays a role in antiviral defense. During the onset of AKI, since the JNK and p38 pathways are closely related to cell stress and apoptosis, the activation of these two pathways by FBXO21 becomes particularly crucial. When activated moderately, it helps to eliminate damaged cells and maintain the stability of the renal internal environment. However, excessive activation leads to an increase in the number of apoptotic renal cells, further exacerbating kidney damage. Jiang et al. (2021) demonstrated that FBXO21 downregulates Nr2f2 to inhibit epithelial-mesenchymal transition (EMT). In the progression of AKI to CKD, EMT plays a key role (Jang et al., 2025). Therefore, the inhibition of EMT by FBXO21 is likely to slow down the transition of AKI to a chronic state, while Lin et al. (2021) found that FBXO21 inhibits autophagy, inflammation, and osteoarthritis (OA) *via* the JUNB-FBXO21-ERK axis. During the onset of AKI, excessive activation of the inflammatory response and imbalance of autophagy levels are common. FBXO21 may protect the kidneys by inhibiting excessive autophagy, alleviating the local inflammatory response in the kidneys, reducing the infiltration of inflammatory cells and the release of inflammatory factors. Although FBXO21 has been extensively studied in fields such as cancer, immune transplantation, anti-fibrosis, and anti-inflammation, reports on its association with AKI are relatively scarce. Thus, it is urgent to deeply explore the specific role of FBXO21 in the pathogenesis of AKI. Subsequent research can start from different stages of AKI onset and deeply analyze its fine-tuning mechanisms of key processes such as autophagy, inflammation, and apoptosis, providing a more solid theoretical basis and potential therapeutic targets for the prevention and treatment of AKI.

Although there is currently a lack of reports on the association between FIZ1 and AKI, this does not imply that there is no connection between them. On the contrary, in-depth exploration of their potential relationship holds great scientific research value. FIZ1 interacts with the catalytic domain of the FLT3 receptor tyrosine kinase (Wolf & Rohrschneider, 1999). FLT3 is a critical therapeutic target, with mutations in FLT3 found in 30% of acute myeloid leukemia (AML) patients, and these mutations are associated with poor prognosis. FLT3 is also implicated in autoimmune diseases (Papaemmanuil et al., 2016). Mali et al. (2008) suggested that FIZ1 may act as a co-regulator of photoreceptor-specific genes, while Larivière, Law & Trinkle-Mulcahy (2014) found that FIZ1 promotes the proliferation of HaCaT immortalized human keratinocytes by activating the MAP/ERK pathway, thereby maintaining epidermal homeostasis. Existing studies on FIZ1 primarily focus on autoimmune diseases, retinal disorders, and epidermal homeostasis. In the pathogenesis of AKI, both autophagy and inflammation play critical roles. Given that FIZ1 interacts with FLT3, it is likely to modulate the downstream signaling network of FLT3, thereby influencing intracellular signal transduction in renal cells. From an autophagy perspective, FIZ1 may regulate autophagosome formation by activating pathways such as MAPK/ERK, either enhancing or inhibiting autophagy in renal cells. This modulation could facilitate the clearance of damaged cellular components or prevent excessive autophagy-induced cell injury, thereby impacting AKI progression. From an inflammatory perspective, FIZ1 may influence the local renal inflammatory response by modulating immune cell activity and altering the secretion of inflammatory mediators, thereby contributing to AKI pathogenesis. These hypotheses, grounded in existing research, underscore the need for further studies to elucidate the precise role of FIZ1 in AKI. Future experimental validation of these mechanisms could provide novel insights into AKI prevention and treatment.

Mast cells are immune cells found throughout the skin and visceral mucosa, where they play a key role in immune regulation by secreting various cytokines that activate T and antigen-presenting cells (APCs) (Breedveld et al., 2017). Zhang et al. (2023) observed a reduced proportion of resting mast cells in diabetic kidney disease (DKD) patients, while Yu et al. (2023b) reported decreased resting mast cells in kidney stone patients. Although mast cells have been studied in the context of hematologic diseases, tumors, allergies, and transplantation, their role in AKI remains underexplored. FBXO21 is a member of the F-box family, and the F-box is part of the four functional domains of Kdm2b (Yan et al., 2018). Lam et al. (2020) found that ELKS1 regulates the transcription of Stxbp2 and Syntaxin 4 by stabilizing Kdm2b, thereby controlling mast cell degranulation. This suggests that FBXO21 may have an impact on mast cells. FIZ1 can bind to the catalytic domain of the FLT3 receptor tyrosine kinase (Wolf & Rohrschneider, 1999), and since FLT3 promotes the proliferation and activation of mast cells (The Cancer Genome Atlas Research Network, 2013), FIZ1 may affect the proliferation and activation of mast cells. In this study, immune infiltration analysis was performed using algorithms, and although this method lacks direct evidence, it suggests that mast cell infiltration may be involved in AKI progression. However, the correlation analysis was based on relatively small sample sizes, and the homogeneity of the samples across different datasets may have introduced bias. To strengthen our conclusions, future studies should employ immunohistochemistry (IHC) or flow cytometry to validate immune cell infiltration in AKI kidney tissues. These approaches would provide direct evidence of immune cell alterations and their spatial distribution in renal tissue, further elucidating their roles in AKI.

However, our research also has some limitations that need to be improved in subsequent work. First of all, the sample size of the transcriptome data set used in this study is relatively limited, which may affect the universality and robustness of the conclusions. Secondly, the cisplatin-induced AKI rat model we adopted has certain representativeness, but AKI itself has multiple causes, including sepsis, ischemia-reperfusion injury, and drug toxicity. Therefore, this model can not completely cover all the causes of AKI, and there is a problem of heterogeneity in causation. Furthermore, although our bioinformatics analysis and animal experiments strongly support the potential of FIZ1 and FBXO21 as biomarkers for AKI, their clinical diagnostic efficacy has not yet been verified in human trials. In the future, it is necessary to carry out prospective clinical trials to evaluate the diagnostic value and application prospects of these two genes in patients with AKI. In addition, it is still unclear how FIZ1 and FBXO21 are specifically involved in the progression of AKI, and in-depth exploration is urgently needed. This includes experimental verification at the cellular level *in vitro* and construction of gene knockout models to further elucidate their functional roles. Finally, the current analysis of immune cell infiltration mainly relies on computational algorithms to estimate, lacking direct experimental evidence to support it. In the next study, we will design specific experiments to address the above issues in order to obtain more conclusive results.

## Conclusions

In conclusion, our study underscores the pivotal role of ARGs in AKI, identifying FIZ1 and FBXO21 as key biomarkers with high diagnostic potential. These genes are involved in critical pathways such as autophagy regulation, calcium signaling, and oxidative phosphorylation, which are essential for kidney function and injury response. The upregulation of FIZ1 and downregulation of FBXO21 in AKI, validated in both bioinformatics analysis and a cisplatin-induced AKI rat model, highlight their significance in disease mechanisms. These findings not only advance our understanding of AKI but also suggest the translational potential of FIZ1 and FBXO21 as early diagnostic markers and therapeutic targets. Future research should focus on validating their diagnostic utility in larger clinical cohorts and exploring therapeutic strategies targeting these genes to improve AKI management and patient outcomes.

##  Supplemental Information

10.7717/peerj.20707/supp-1 Supplemental Information 1 Code

10.7717/peerj.20707/supp-2 Supplemental Information 2 Western blots

10.7717/peerj.20707/supp-3 Supplemental Information 3 Renal index, BUN, SCR, weight

10.7717/peerj.20707/supp-4 Supplemental Information 4 Supplemental tables

10.7717/peerj.20707/supp-5 Supplemental Information 5 ARRIVE Checklist

## Additional Information and Declarations

### Data Availability

The following information was supplied regarding data availability: The data is available in the Supplemental Files. The raw sequence data are available at NCBI BioProject: GSE61739, GSE139061, GSE53769
