# Supplementary figures and images for: Comprehensive machine learning and experimental verification reveal the mechanism of action of autophagy-related genes FIZ1 and FBXO21 in acute kidney injury
Source: PeerJ. 2026 Feb 2;14:e20707. doi: 10.7717/peerj.20707 (PMC12875250; doi:10.7717/peerj.20707)

FBXO21

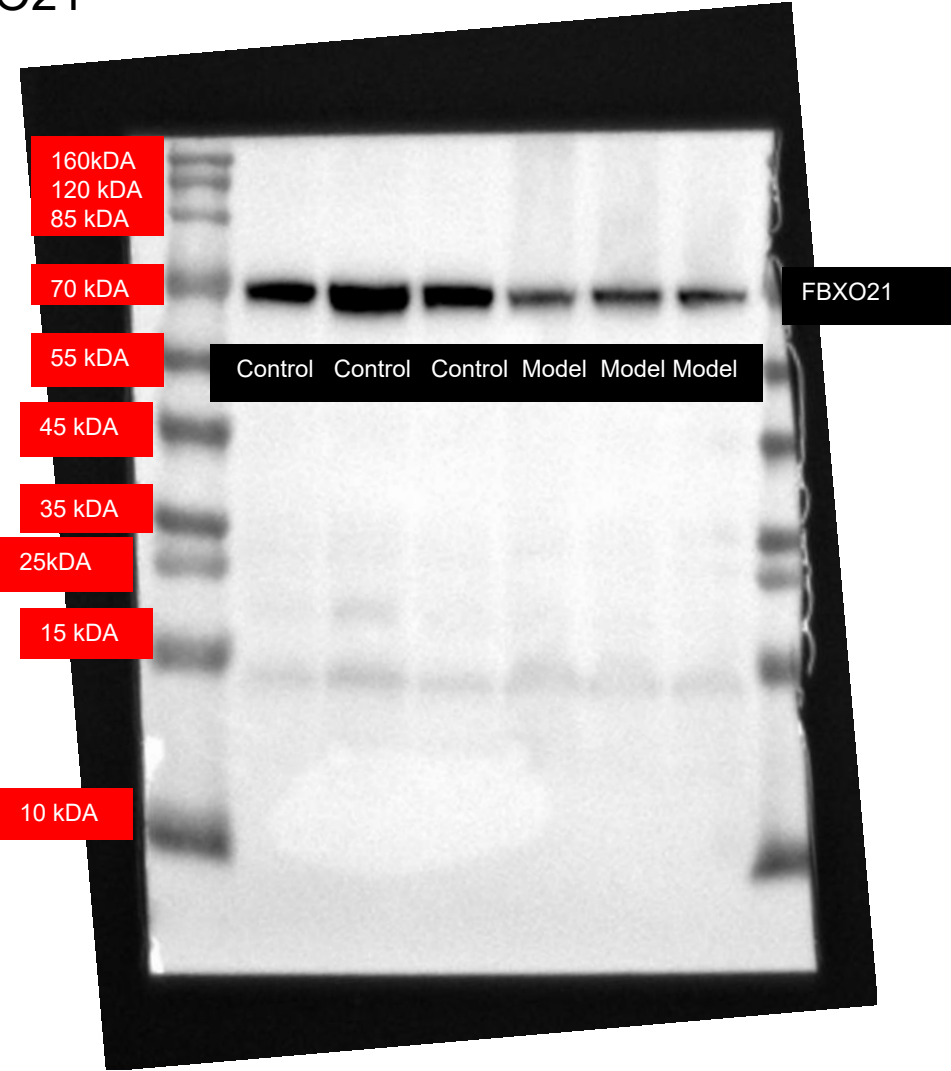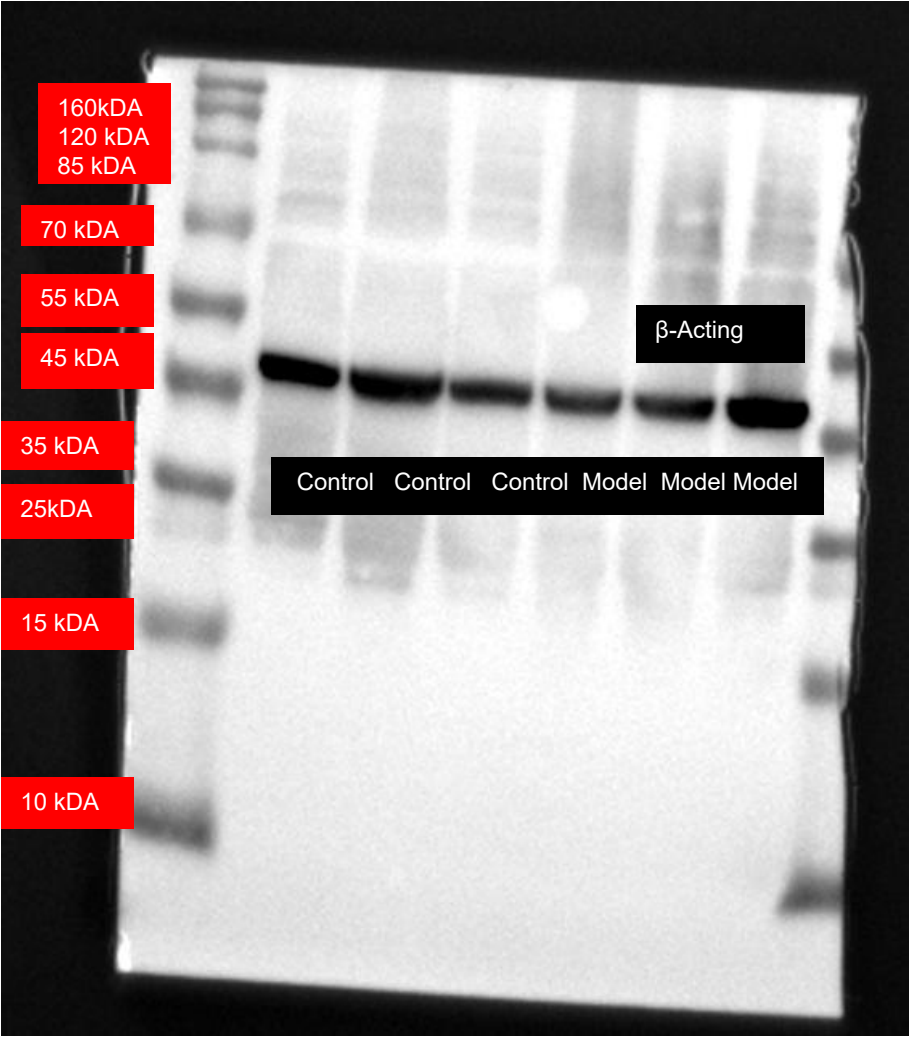

FBXO21

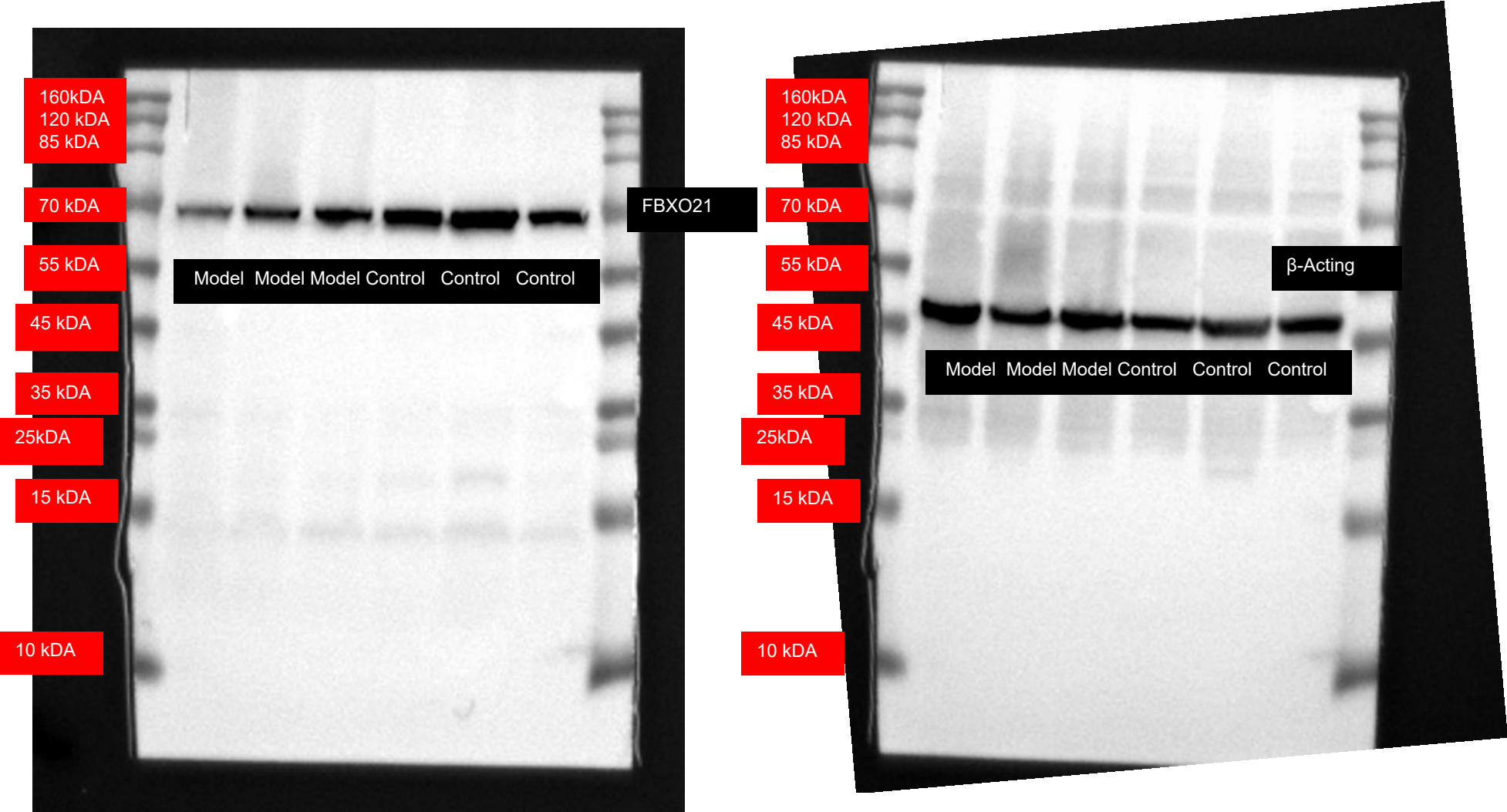

FBXO21

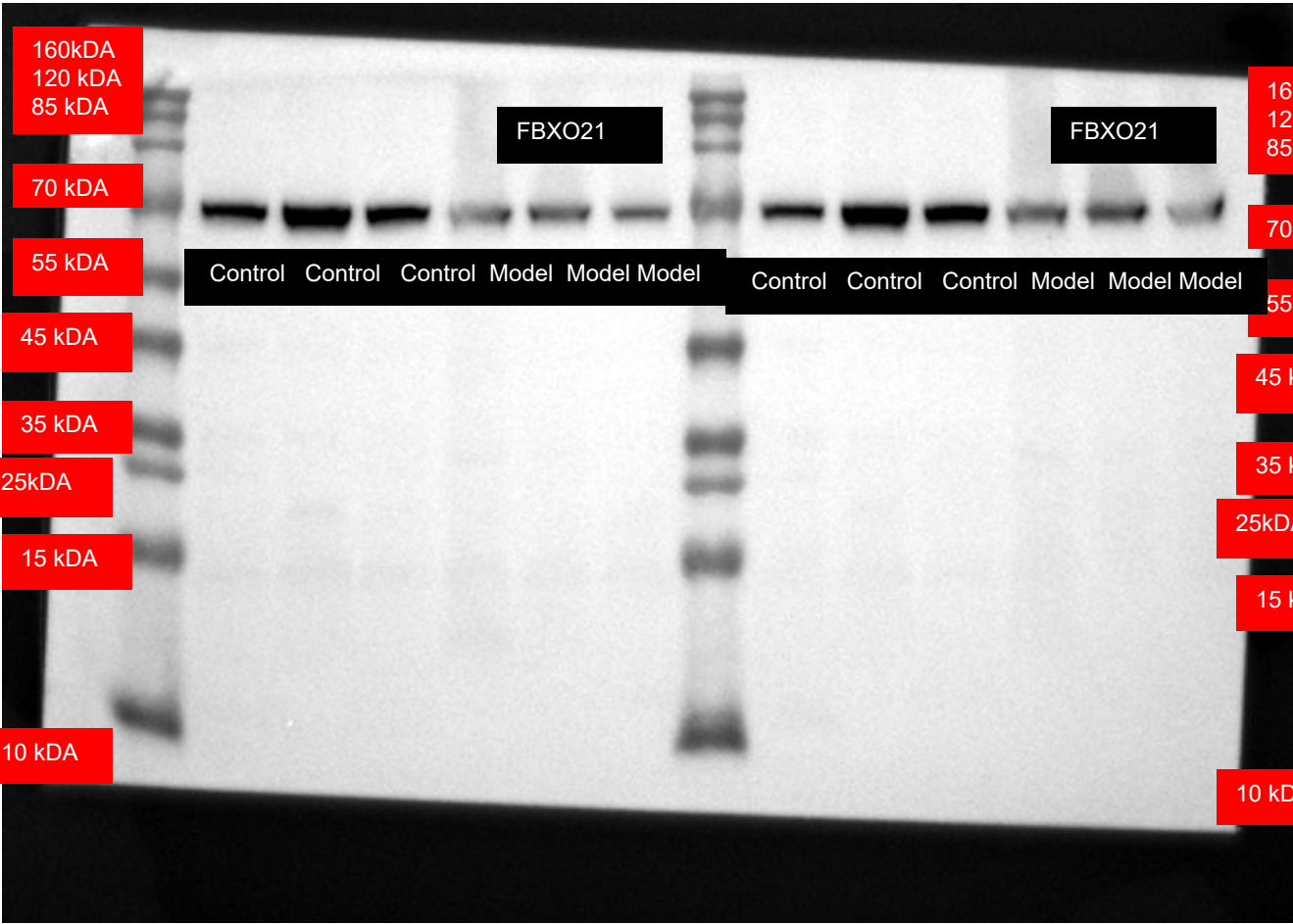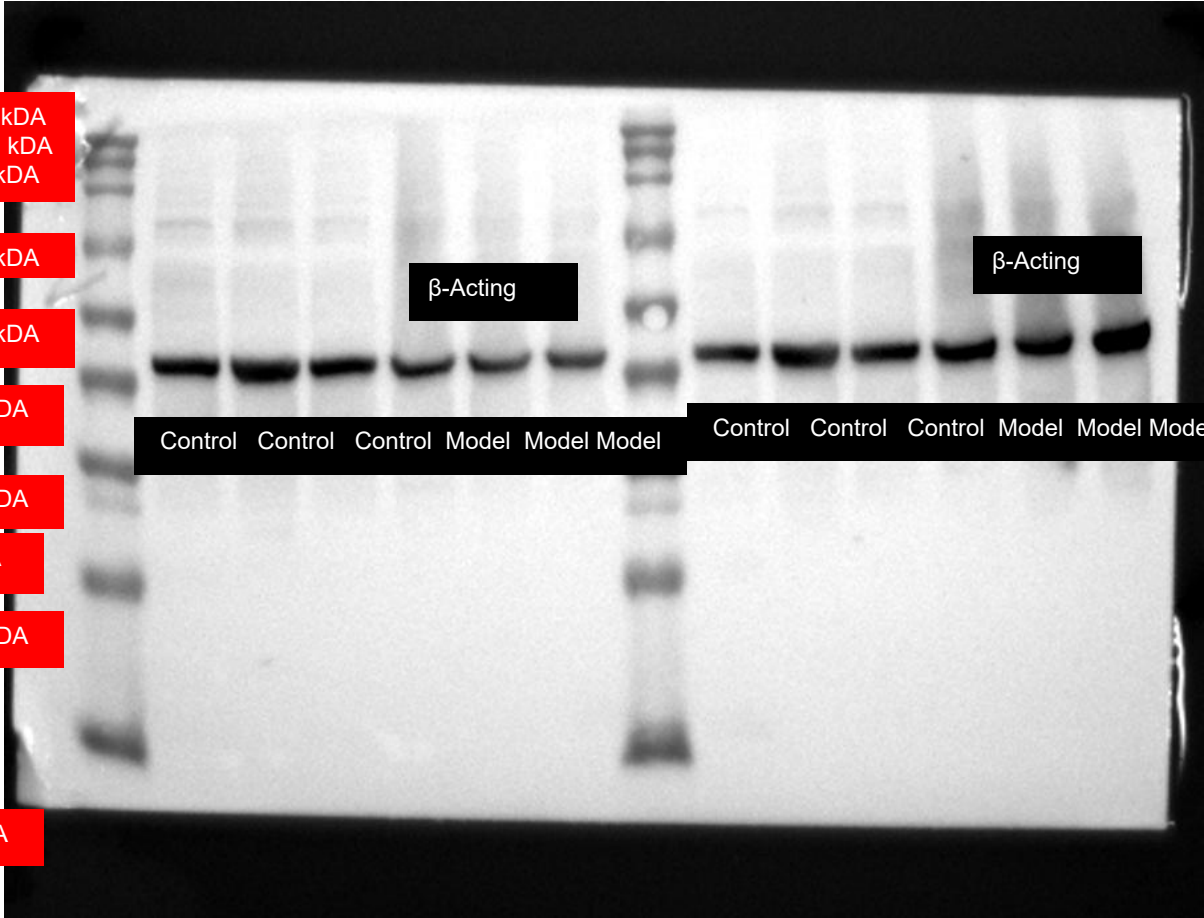

FIZ1

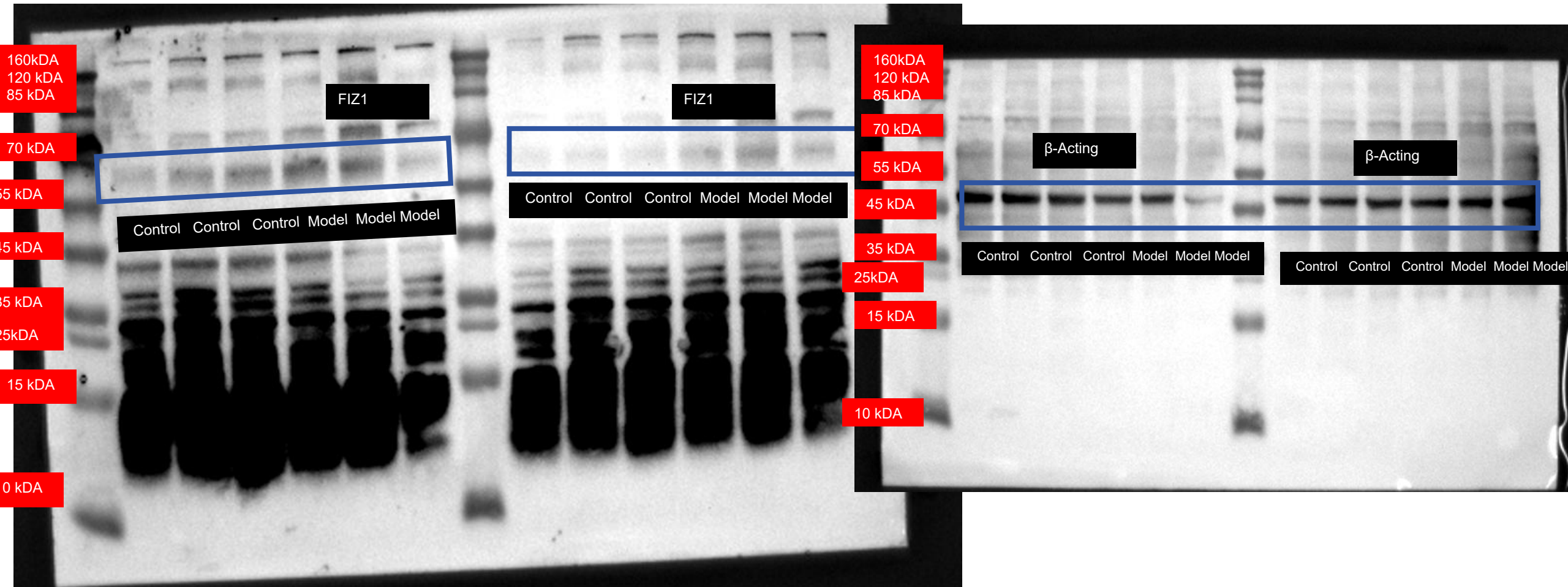

FIZ1

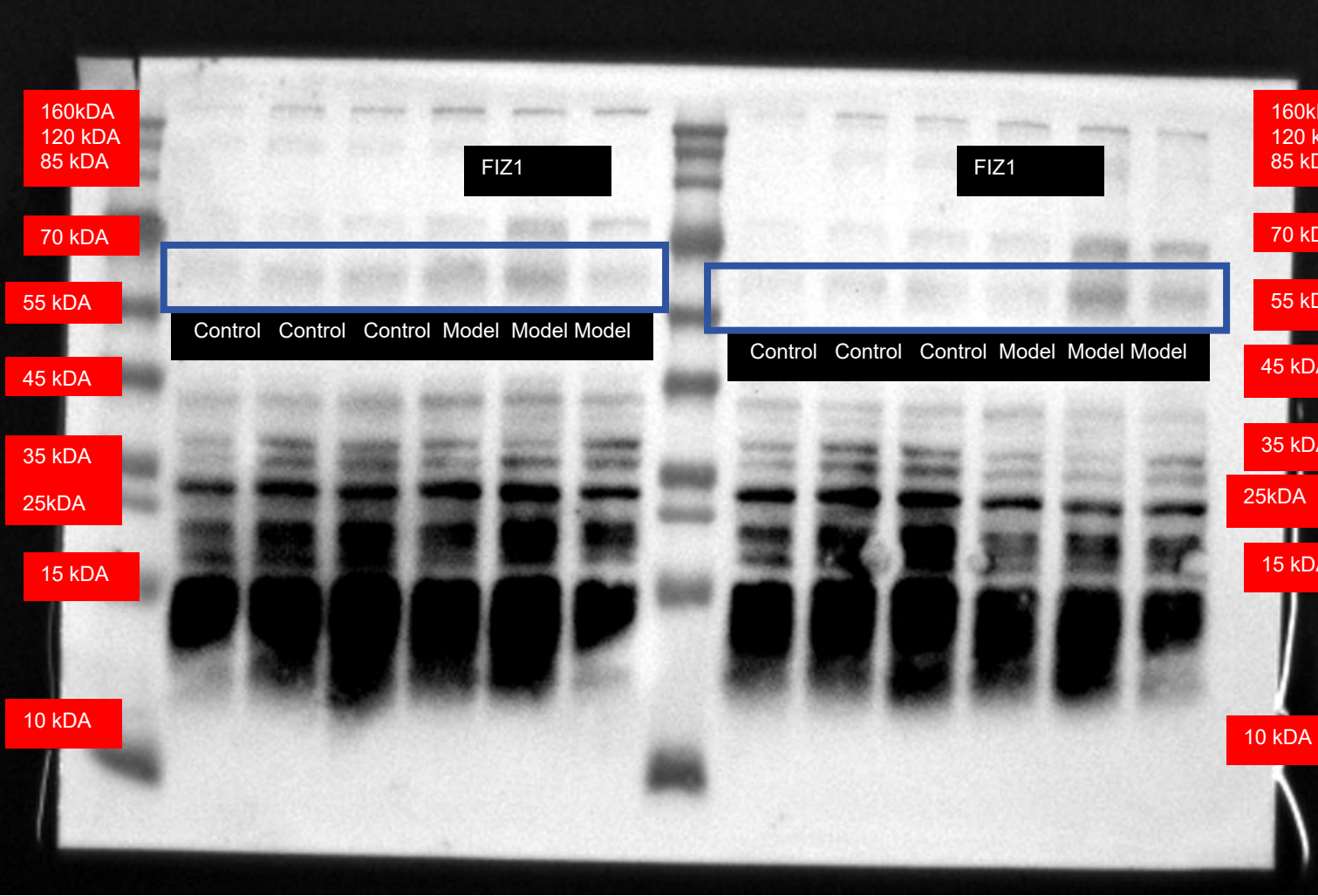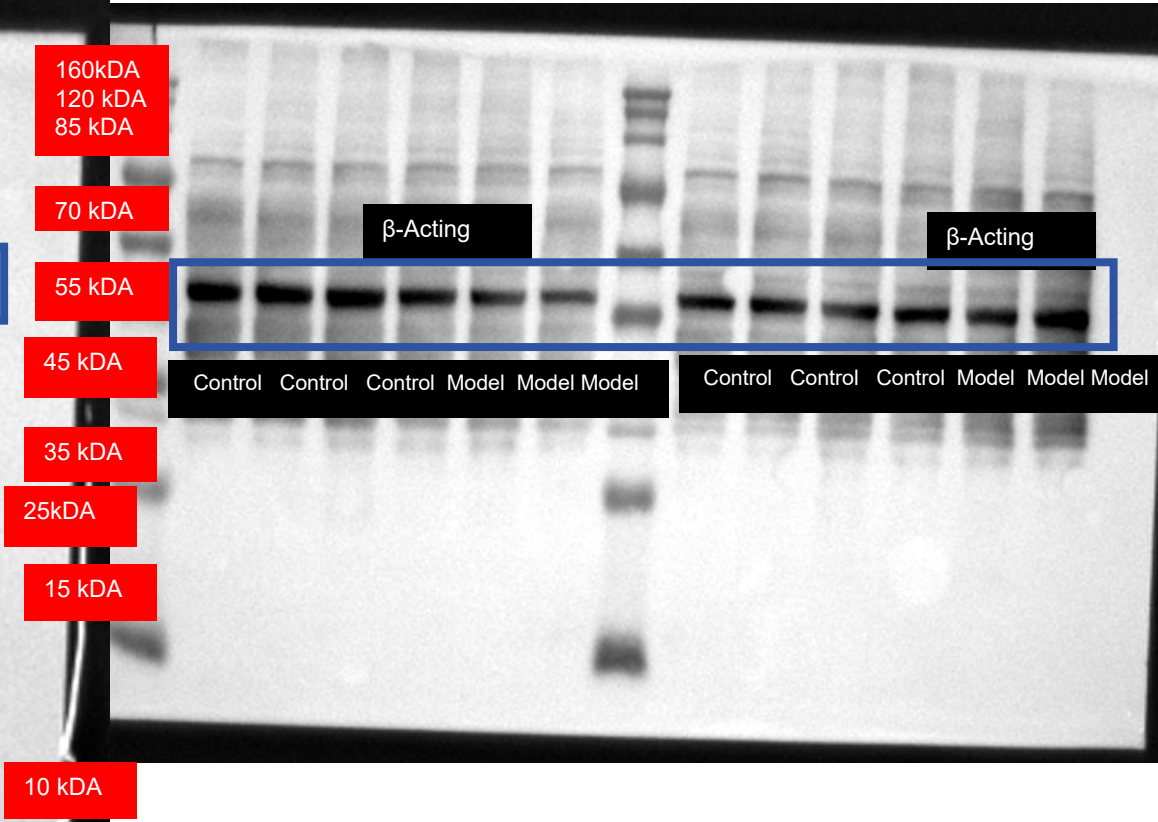

Supplement: Supplemental Information 2 [file peerj-14-20707-s002.pdf]
