# Supplementary material for: Comprehensive machine learning and experimental verification reveal the mechanism of action of autophagy-related genes FIZ1 and FBXO21 in acute kidney injury
Source: PeerJ. 2026 Feb 2;14:e20707. doi: 10.7717/peerj.20707 (PMC12875250; doi:10.7717/peerj.20707)
Supplement: Supplemental Information 4 [file peerj-14-20707-s004.docx]

**Supporting Information**

Comprehensive Machine Learning and Experimental Verification Reveal the Mechanism of Action of Autophagy-Related Genes FIZ1 and FBXO21 in Acute Kidney Injury

Yunqi Bai^1, 2, a^, Lili Zhang^2, a^, Bo Nie^1,a^,Yixin Su^1, 2, *^, Jingwei Zhou^1, *^

1 Beijing University of Chinese Medicine First Affiliated Hospital, Beijing 100700, China

2 Beijing University of Chinese Medicine, Beijing 100029, China

**Corresponding Author**

* Yixin Su, Department of Nephrology, Dongzhimen Hospital, Beijing University of Chinese Medicine, Beijing 100700, China; Phone: 86-18511822517; suchangfriend@sina.com

* Jingwei Zhou, Beijing University of Chinese Medicine, Beijing 100029, China; Beijing University of Chinese Medicine First Affiliated Hospital, Beijing 100700, China; Phone: 86-13910634708; [13910634708@163.com](mailto:13910634708@163.com)

a These authors contributed equally to this work.

**Table S1.The HADb database provides 232 ARGs**

| AMBRA1 | CTSB | CHMP2B | HIF1A | BECN1 | FKBP1B | PEX14 | SESN2 | ATG4A | EEF2 | NBR1 | ITPR1 |
| --- | --- | --- | --- | --- | --- | --- | --- | --- | --- | --- | --- |
| APOL1 | CTSD | CHMP4B | HSP90AB1 | BID | FOS | PEX3 | SH3GLB1 | ATG4B | EEF2K | NCKAP1 | KIAA0226 |
| ARNT | CTSL1 | CLN3 | HSPA5 | BIRC5 | FOXO1 | PIK3C3 | SIRT1 | ATG4C | EGFR | NFE2L2 | KIAA0652 |
| ARSA | CX3CL1 | MAPK3 | HSPA8 | BIRC6 | FOXO3 | PIK3R4 | SIRT2 | ATG4D | EIF2AK2 | NFKB1 | KIAA0831 |
| ARSB | CXCR4 | MAPK8 | HSPB8 | BNIP1 | GAA | PINK1 | SPHK1 | ATG5 | EIF2AK3 | NKX2-3 | KIF5B |
| ATF4 | DAPK1 | MAPK8IP1 | IFNG | BNIP3 | GAA | PPP1R15A | SPNS1 | ATG7 | EIF2S1 | NLRC4 | KLHL24 |
| ATF6 | DAPK2 | MAPK9 | IKBKB | BNIP3L | GABARAP | PRKAB1 | SQSTM1 | ATG9A | EIF4EBP1 | NPC1 | LAMP1 |
| ATG10 | DDIT3 | MBTPS2 | IKBKE | C12orf44 | GABARAP | PRKAR1A | ST13 | ATG9B | EIF4G1 | NRG1 | LAMP2 |
| ATG12 | DIRAS3 | MLST8 | IL24 | C17orf88 | GABARAPL1 | PRKCD | STK11 | ATIC | ERBB2 | NRG2 | MAP1LC3A |
| ATG16L1 | DLC1 | MTMR14 | IRGM | CALCOCO2 | GABARAPL1 | PRKCQ | TBK1 | BAG1 | ERN1 | NRG3 | MAP1LC3B |
| ATG16L2 | DNAJB1 | MTOR | ITGA3 | CAMKK2 | GABARAPL2 | PTEN | TM9SF1 | BAG3 | ERO1L | P4HB | MAP1LC3C |
| ATG2A | DNAJB9 | MYC | ITGA6 | CANX | GABARAPL2 | PTK6 | TMEM49 | BAK1 | FADD | PARK2 | MAP2K7 |
| ATG2B | DRAM1 | NAF1 | ITGB1 | CAPN1 | GAPDH | RAB11A | TMEM74 | BAX | FAM48A | PARP1 | MAPK1 |
| ATG3 | EDEM1 | NAMPT | ITGB4 | CAPN10 | GAPDH | RAB1A | TNFSF10 | BCL2 | FAS | PEA15 | SAR1A |
| CAPN2 | GNAI3 | RAB24 | TP53 | CCR2 | GRID1 | RB1CC1 | ULK1 | BCL2L1 | FKBP1A | PELP1 | SERPINA1 |
| CAPNS1 | GNAI3 | RAB33B | TP53INP2 | CD46 | GRID2 | RELA | ULK2 | CASP3 | GNB2L1 | RAB7A | TP73 |
| CASP1 | GNB2L1 | RAB5A | TP63 | CDKN1A | GRID2 | RGS19 | ULK3 | CASP4 | GOPC | RAC1 | TSC1 |
| CASP8 | GOPC | RAF1 | TSC2 | CFLAR | HGS | RPTOR | VAMP3 | CDKN1B | HDAC1 | RHEB | USP10 |
| CCL2 | GRID1 | RB1 | TUSC1 | WIPI1 | VEGFA | WDR45L | VAMP7 | CDKN2A | HDAC6 | RPS6KB1 | UVRAG |
| WIPI2 | WDFY3 | ZFYVE1 | WDR45 |  |  |  |  |  |  |  |  |

**Table S2.A total of 4185 ARGs**

| VMA21 | RECQL | BIRC3 | EPHB6 | WNK1 | PVR | MIR150 | TMCO1 | PSMC5 | ATP6V0A1 | PSMD11 | MIR1301 |
| --- | --- | --- | --- | --- | --- | --- | --- | --- | --- | --- | --- |
| ATG5 | P3H1 | GDF15 | NOL3 | CCDC115 | POFUT1 | TMEM192 | ZDHHC5 | LMNA | HAVCR1 | ALKBH5 | AQP5 |
| BECN1 | NR2F1-AS1 | MIR96 | SLC39A4 | CHMP2A | RCC1 | ING2 | DCAF7 | MIR376B | PGK2 | SUPT16H | KIF22 |
| ULK1 | PGR | FTH1 | TRAF5 | TXN | SLC39A14 | RCN2 | MMS19 | RIPK1 | NAA15 | RPS3A | LLGL1 |
| ATG16L1 | PLA2G4A | BICD2 | RAB3A | HTRA2 | NPEPPS | NAP1L1 | QSOX2 | IGF1 | VPS13B | DDIT4 | MIR491 |
| ATG7 | TLR7 | ATP6AP2 | SMPD2 | YY1 | USF1 | FH | SYNGR2 | CXCR4 | ST13 | PRDX2 | CLPB |
| ATG14 | PEBP1 | RPS2 | CHRD | EIF4EBP1 | GIPC1 | ABCD3 | NENF | RPS27A | CAB39 | VTI1B | GPX1 |
| AMBRA1 | MIR222 | UBTF | EPHA8 | EIF2AK4 | PPAT | RPL37A | MED19 | SNAP29 | URB2 | TRAPPC1 | AKAP8 |
| ATG12 | PELO | KRT18 | IL27RA | MIR142 | TIMM44 | ILF3 | RETSAT | VPS18 | YLPM1 | FARSB | MSH2 |
| ATG4B | CC2D1A | EMD | ITGA11 | RELA | KXD1 | MIR372 | MRPL21 | LEP | MIR424 | TNFRSF11B | CASP10 |
| SQSTM1 | MDC1 | MARCHF7 | ITGAE | PEX5 | UGGT1 | GFPT1 | EMC7 | BRAF | EFNB2 | CAPRIN1 | LTBP1 |
| ATG13 | DDX23 | TRIM59 | ITGB8 | CASP1 | CRMA | TOP2B | MIR454 | PGR-AS1 | BMP6 | ZDHHC19 | NRP2 |
| ATG3 | CAPZB | TTN | EPHA6 | IGF1R | SNCAIP | RIMOC1 | CA2 | UBE2D3 | RAB27B | MT-TP | NDUFS8 |
| ATG9A | TRIM7 | CALR | NPAS2 | ATP5F1A | FLG2 | XPOT | RUNX1 | MDM2 | SIVA1 | BCL2A1 | IDH3A |
| ATG4A | SCN5A | ESR1 | TNFRSF10C | FNBP1L | ZFPM2-AS1 | SERBP1 | APPL1 | XBP1 | RAB40C | TFAP2C | MAGT1 |
| ATG4D | BCAR1 | MIR93 | ITGAD | NEK1 | SNX1 | SERPINB5 | SLC38A1 | IL23R | RAB40A | BSG | L2HGDH |
| ATG4C | CD63 | CCR5 | ITGB3BP | IMMT | MON1B | SLC39A1 | PDLIM7 | PSMA6 | YME1L1 | ACLY | HDGF |
| ATG10 | SDC2 | RPLP0 | MEPE | INS | CRYAB | PPIA | SNX10 | RIPK2 | PTPN6 | CDK7 | ANKFY1 |
| EPG5 | CUL2 | MIR195 | MNDA | NEDD4 | GTF2I | RPL13 | GSDMA | VPS33A | NACC1 | C1QBP | SLC30A7 |
| ATG9B | ACSL3 | KDM1A | RAB28 | ATP12A | RPS15 | ZFYVE26 | ARL8A | TRIM16 | MIR138-1 | RPS6 | WASHC4 |
| MAP1LC3B | TCOF1 | MIR181A2 | GIMAP5 | ACER2 | BMP2K | RPL11 | MIR122 | VDAC1 | TBC1D4 | ERLIN2 | CIAPIN1 |
| ATG2B | SLC38A2 | PPP2R1A | CXCL14 | DANCR | SAP18 | HUNK | MIR376C | KAT5 | RUSC2 | RPS9 | TAMM41 |
| ULK2 | ARPC4 | PSMD3 | EDIL3 | KCNQ1OT1 | MECP2 | OSBPL8 | MYBPC3 | TSC1 | SERPINB12 | PIN1 | GOLGB1 |
| MAP1LC3A | UBA3 | CCND1 | ICAM4 | BCAP31 | ENG | CCDC47 | ATXN10 | PHB2 | H2BC15 | SBF2 | NDC1 |
| DRAM1 | WDR74 | HADHA | ITGA10 | USP20 | CCN2 | HLA-DRB5 | CHCHD2 | ATXN2 | ABL2 | MAGEA3 | ECPAS |
| ATG101 | PAPPA-AS1 | TRIM28 | MXD1 | RAB39A | RELB | DELEC1 | NIPA1 | TMEM74 | MADD | UQCRC2 | TRABD |
| MTOR | LOC126863256 | MIR301B | NDRG4 | NLRP1 | MID1 | EI24P1 | GPI | VPS54 | MARK2 | DVL2 | CREB3 |
| DRAM2 | FOXE3 | CKB | EMB | PABPC1 | HDLBP | EI24P2 | IRF2 | DNM1L | TIMD4 | ACACA | PMPCB |
| ATG16L2 | NDUFV1 | ZRANB1 | IL9R | RRAGC | PIEZO1 | EI24P4 | MYO5A | CAV1 | LIX1 | CLINT1 | NAPG |
| NBR1 | TOR1AIP1 | TUBB4A | NDRG3 | FN1 | RASAL2 | EI24P3 | SH3GL1 | HSF1 | CTNND1 | IL4 | USP12 |
| ATG2A | GLI1 | SLK | SLC39A3 | ATP2A2 | BLZF1 | EI24P5 | MYL6B | PSEN2 | FEN1 | AP2A1 | FLAD1 |
| LAMP2 | TAFAZZIN | ATXN7 | IL20RB | BANCR | SPATS2L | VDR | LIMCH1 | PRKAG1 | TGFBI | ACAT1 | CXCR3 |
| RUBCN | DNAJC10 | EPHA2 | MVB12B | PDCD4 | WASHC2A | EIF2S3 | CISD1 | TRIM17 | SPHK2 | CTSS | FNDC5 |
| RUBCNL | ULK4 | JAK1 | RAB40B | EIF5A | LINC00515 | HNRNPF | DNMT3A | DAO | CHAF1B | GNAI3 | KLF9 |
| BCL2 | TRAPPC6B | RPL23A | ATRAID | RAB21 | MIR376A1 | BAG1 | ADAMTS9-AS2 | PIK3CB | TRIM14 | HGS | POR |
| RB1CC1 | MTCL1 | ORMDL3 | RAB3B | RRAGD | RRAS2 | FBXW11 | ADAM17 | LGALS3 | VPS26B | PRPF19 | HSPG2 |
| GABARAPL1 | BDNF | HNRNPC | PHLDA3 | PRKAR1B | WRN | ELOB | CSNK1D | PHAF1 | MIR107 | SEC61A1 | ALPI |
| MAP1LC3C | EPS15 | TP73 | SLC39A2 | TMPO | MCM6 | GPX4 | CSK | SMAD5-AS1 | EPS15L1 | RPS5 | F13A1 |
| GABARAP | ABCB1 | DHX9 | RAB22A | SNORD15A | USP28 | RNY5 | DDB2 | DIRAS3 | DYSF | MIRLET7B | LPIN1 |
| PIK3C3 | KLF5 | ARL13B | RAB3C | SESN2 | DNAJB12 | RAP1A | GNAI2 | UBE2D2 | TBC1D9 | EPHA1 | LAMC1 |
| DEPP1 | LRG1 | RUVBL2 | RAB4B | PGAM5 | CORO7 | LITAF | HSD17B4 | BIRC5 | SEH1L | IL15 | MYL1 |
| ELAPOR1 | TBC1D2 | PCM1 | RAB20 | BCAS3 | SPECC1 | STAU1 | NME1 | NPC2 | DYNLT1 | RAB30 | JMJD6 |
| EI24 | MIR31 | PSMC6 | RAB6C | MFN1 | UBXN1 | MIR410 | S1PR1 | CDKN1A | LINC00958 | TMEM43 | CSRP1 |
| TFEB | PPP3CA | RAB5B | ALKBH7 | EPHB2 | PTGES3 | MST1 | SLC12A6 | BAD | AP4B1 | IL13 | POLR3D |
| GABARAPL2 | FANCC | MTHFD1 | PIK3R6 | BSN | CPOX | CSE1L | GNA13 | YWHAZ | PRKCE | TBC1D1 | RIOK2 |
| HMGB1 | CTPS1 | IGF2R | RAB40AL | HMGA1 | PPP4C | PABPC4 | KIF2A | HEXB | SPINK1 | RUVBL1 | SRP68 |
| OPTN | PGRMC1 | MIR30E | RNF103-CHMP3 | BAK1 | SRI | RPL14 | CBR1 | PSMC2 | MUC1 | KL | ARHGAP10 |
| FYCO1 | IQGAP1 | TIAL1 | ESD | SUPT20H | KISS1 | SSBP1 | COQ6 | TOLLIP | SOX2 | INA | CTNNBL1 |
| UVRAG | SLC25A22 | DCP2 | RARRES1 | PLEKHM2 | KLRK1 | DYNC1I2 | DUSP3 | YWHAE | MARS2 | FBXL2 | DCD |
| IRGM | ATAD3A | NSF | RNF135 | ELP3 | YEATS4 | GARS1 | EED | PKM | NUP188 | RPSA | METTL13 |
| TBK1 | PDIA2 | IAPP | UBE2M | VDAC3 | LINC00861 | PDE4A | EXOSC3 | PSMA1 | TMEM30A | AHCY | DMAC2 |
| PRKAA1 | CARM1 | SKP2 | ANGPTL8 | ITPR1 | LRBA | SLC7A5 | UBR1 | RETREG1 | AQR | SSRP1 | CASP8AP2 |
| TP53 | MIR197 | MIR193A | RAD50 | TUBA1C | RPL26 | PIP4K2C | DCXR | TMEM41B | BANP | RPL18 | LINC00472 |
| VCP | MIR1-1 | TRIB3 | NANS | VHL | SEC63 | NCOR1 | GNB4 | CFTR | USO1 | MIR221 | RINT1 |
| C9orf72 | FBXL3 | PRPF8 | DNAJB6 | CCAR2 | RPL27A | MIR29B1 | GPAA1 | CSF1R | TSR2 | FIS1 | SPATS2 |
| WIPI1 | ALK | TRAF4 | HAT1 | DDIT3 | AUP1 | PLEKHG5 | SLC4A2 | MAPK15 | LGALS7 | EXOSC2 | GLI3 |
| PIK3R4 | SEC24A | MIR223 | TMOD3 | TNFSF11 | MTX1 | FTO | PCYT2 | NOTCH3 | RPL26L1 | LONP1 | GLI2 |
| MIR7-3HG | CDK2 | HIPK2 | RBM15 | RAB5C | RPL36A | CIP2A | PITRM1 | CCNF | MIR129-2 | MIR889 | SPTLC2 |
| ATG12P1 | ARF4 | PIK3CD | OCIAD2 | FOXK2 | COPS8 | ENPP1 | ARPC3 | LOC129995449 | CNOT4 | MIR335 | TGM1 |
| ELAPOR2 | CALCOCO1 | DDX17 | NIFK | RSL1D1 | SART1 | CAT | CEP63 | BNIP1 | PRKCZ | PRDX1 | BLVRA |
| WIPI2 | TNFAIP8L2 | TRADD | MT-TK | JAK2 | HEXIM1 | STT3A | ETS2 | FUNDC1 | ECHS1 | MIR15B | MAPKAP1 |
| HDAC6 | HSPA6 | HERC1 | ELAVL4 | CLEC16A | NOTCH2 | GLG1 | PPA2 | RAB11A | SPTBN1 | LINC00511 | HAL |
| VMP1 | SLC25A4 | KCMF1 | ECE1 | TNFRSF10B | PLN | MTCH2 | SF3B2 | DNAH8 | CDC34 | ACO2 | RBBP4 |
| ATG3P1 | ARF1 | FLT3 | ARHGAP1 | LARP1 | FSTL1 | PTGS2 | SNX5 | TIGAR | SARS1 | PPARGC1A | DEGS1 |
| ATG12P2 | HSPE1 | UCA1 | GOSR2 | RNU6-1 | TRPC5 | UHRF1 | EGFL7 | MEFV | PREP | RPL3 | IARS2 |
| CALCOCO2 | DRG1 | ZFAS1 | PEX6 | USP14 | HYPK | HLA-A | RNPEP | NIPSNAP1 | SRP72 | RPS7 | SRSF9 |
| PRKN | PFKL | RAB6B | AP3M1 | EIF2S1 | PIP4K2B | EGR1 | SDF4 | CHCHD10 | PKP3 | GDI2 | PPIL2 |
| HSPA8 | AMFR | TEX264 | TGOLN2 | BIRC2 | PDE3A | PTPA | WASHC5 | RRAGA | MRPL38 | CTSC | UGGT2 |
| UBQLN2 | PEX3 | PYCARD | ADGRA3 | HEXA | TAP1 | ATN1 | ZC3H14 | MIR155 | PTOV1 | TWNK | ADPGK |
| PARK7 | POLR2C | KAT8 | PTPMT1 | MAP2K1 | MAPKAPK3 | DEK | CENPB | HSP90AB1 | HDHD5 | EPRS1 | TUT1 |
| ATG4AP1 | IVNS1ABP | SLC25A13 | RPL38 | APOB | SIN3A | SNRNP200 | NDFIP1 | HAGLROS | H2AC14 | FASLG | GULP1 |
| AKT1 | OTUB1 | DDX1 | WDR82 | HDAC7 | GALNT2 | RABGAP1 | NEMF | TFE3 | MIR128-1 | NUP160 | VPS35L |
| SCARNA5 | PSMD1 | FKBP4 | PLBD2 | OGT | LATS1 | WDR1 | SLC16A4 | LOC109504728 | MIR665 | RGS19 | DNAJC16 |
| SNCA | CSRP2 | SPRED2 | KBTBD6 | RIPK3 | NRF1 | MGMT | TAF1C | IFI16 | ITSN1 | MIR4458 | DDC |
| MTCL2 | MESD | SLC2A8 | MAP4K2 | MAP3K7 | PSMB1 | H3C1 | EIF3I | UBE2N | MCM3 | MYO1B | EDC4 |
| SIRT1 | UNC45A | RBMX | SAMHD1 | TBC1D17 | ANP32A | METTL14 | EDEM3 | GSK3A | FAM120A | TRP-AGG2-6 | DGCR5 |
| WDFY3 | NUDCD3 | MIR146A | WNK4 | ERBB4 | GHRL | EEF1A2 | ITM2A | NIPSNAP2 | PRKD1 | CCPG1 | MIR493 |
| PRKAA2 | LANCL2 | DNAJA1 | HACE1 | TBP | DCAF1 | LETM1 | NCEH1 | TREM2 | CTSZ | NCOA3 | NBAT1 |
| NFE2L2 | CAMK2A | MIR338 | KLHL3 | CHRM1 | PSME1 | AGK | PDS5A | TOM1 | NOX4 | RPL8 | MRPL41 |
| SOD2-OT1 | KDM3B | FGF21 | USP18 | ABCA7 | UBAP2L | BRCA1 | RO60 | SPART | LGALS3BP | VPS45 | MIR138-2 |
| LOC727709 | LATS2 | CASC9 | ENAH | DPP6 | RDH13 | HSD17B10 | DNAJC2 | NEDD4L | E2F4 | MIR29A | HNF4A |
| PINK1 | PHLDA2 | RPS16 | BCL2L12 | PON3 | ZWINT | RALB | GPN1 | TNFAIP3 | GSTM3 | UBXN6 | RDX |
| H19 | LYZ | SLC52A3 | ZFPL1 | TYROBP | MRPL49 | RRM1 | NEDD1 | SNCB | RBCK1 | SMC3 | FBLN1 |
| SH3GLB1 | CYB5R3 | RRAS | FBXL20 | TIMM8A | DDX27 | AIF1 | SRSF5 | CASP9 | CLEC4A | HADHB | GJA8 |
| WDR45 | TMIGD2 | REEP1 | MIR218-1 | SRCAP | TIPRL | CCNY | ARID3A | ANG | MIR139 | MIR129-1 | AOC1 |
| RPTOR | TLX1NB | SLC52A2 | MIR637 | SS18L1 | MIR99A | TMEM33 | DERA | YAP1 | MIR377 | PGD | LMO2 |
| LINC01672 | PIP5K1C | SCAF4 | DARS1-AS1 | ARHGEF28 | NFKB2 | PSMD12 | GTF3A | PIM1 | SRP14 | PNO1 | PAPPA |
| NOD2 | ARL1 | TROAP-AS1 | LOC130002815 | FGGY | ACTA1 | NEDD8 | COPS4 | DYNC1H1 | GLB1 | ENO1 | TPP2 |
| FOXO3 | MIR203A | SOD1-DT | LINC03039 | LOC129929032 | RBM10 | SRSF2 | ERGIC2 | ABL1 | SLC2A3 | KIF5B | JMJD1C |
| BNIP3 | TARS1 | LOC124629354 | MIR519D | MCOLN3 | H1-5 | PTPN2 | TRIAP1 | AIFM1 | NR1H2 | JUP | PHF6 |
| CERNA3 | CYFIP1 | RPS23 | ATP6V0B | HDAC8 | RBBP6 | IL10RB | ZRANB2 | BOK | GPC4 | RPS27 | UPF3B |
| BDNF-AS | NCAPD2 | FUNDC2 | SCARB1 | UBE2V1 | G3BP2 | KLHL22 | FOXRED2 | AIM2 | BRSK2 | MTMR2 | AKAP12 |
| LRRK2 | DACT1 | BRD4 | PRPS1 | DYNLL2 | SRP9 | TOP1 | TMEM259 | ZFYVE1 | SMCHD1 | ACTN4 | DHX8 |
| UBQLN1 | TRMT10C | MIR4487 | ARRB2 | TUBB3 | COL3A1 | PSMA2 | LRRC47 | ATP4A | S100A7 | BAG2 | ECI1 |
| KEAP1 | SACM1L | PTPN11 | RAP1B | MITF | KMT2A | PELP1 | RNF217-AS1 | GBE1 | GPX8 | PML | OBSL1 |
| MAPT | IDH1-AS1 | DDX6 | PXDN | EIF4G2 | RORB | TECR | LINC00920 | IL10RA | CPVL | SLC25A12 | RBM17 |
| UBC | CDK16 | STBD1 | ORC4 | NCL | SFN | HLA-DQB1 | LINC01215 | RAB33B | LANCL1 | HBB | TSR1 |
| BECN2 | VPS4B | ATP6V1E1 | DCTN2 | MIR320A | IL12RB2 | IL37 | DMPK | AGER | ZG16B | TUBAL3 | ARHGEF11 |
| TP53INP2 | DDX39B | ATP5F1B | SWAP70 | TNFRSF1A | IL21 | SNHG7 | SGSH | ANXA11 | SEC61A2 | ATP6V0D1 | NSUN5 |
| TARDBP | CS | RPS28 | SRSF10 | CSNK2B | STK24 | IDO1 | SERPINI1 | PON2 | LINC00857 | RPS19 | SVEP1 |
| DAPK1 | VAMP3 | TMEM106B | BLOC1S2 | TRA-TGC7-1 | MLX | FABP5 | LARP7 | EIF2AK3 | PIGS | RPL13A | DDX50 |
| STAT3 | TMX1 | CHMP6 | TOR1AIP2 | ABCE1 | TNFRSF6B | NOS2 | RHBDF1 | BCL2L11 | MMP9 | EIF3A | PGLS |
| TAX1BP1 | LINC02527 | MIR381 | UTP15 | ERBB2 | TRAF1 | SIRT7 | ANKDD1A | LAMTOR1 | PLOD3 | RANGAP1 | IGHG1 |
| RAB7A | IL12B | FBP1 | DHX33 | MTDH | CTHRC1 | TNFAIP8L1 | LINC01389 | IFNB1 | GGPS1 | THORLNC | ZFAT |
| TP53INP1 | BCR | AZI2 | CCDC124 | HNRNPK | IL15RA | IL3 | LRP1-AS | MIR125A | ZFP36 | PLAC8 | ARHGEF19 |
| TMX2-CTNND1 | CBL | IKBKG | MIR874 | PTPN22 | ZC3HC1 | ASS1 | MIR4673 | BSCL2 | TM9SF2 | AP2B1 | GMCL1 |
| STX17 | TGFB2 | IMPDH2 | LPL | CSNK2A2 | ITGB1BP1 | IRS4 | ZEB1 | TRIM23 | BORCS6 | NQO1 | MAP7D3 |
| HSP90AA1 | ERCC2 | DNAJB1 | ANK3 | MIR34C | RFC1 | RPL15 | CTCF | AURKB | ERMP1 | WNT16 | DCAF5 |
| WDR41 | BUB3 | CDC37 | CTTN | BIK | SYVN1 | TRIP13 | NR3C2 | MTMR3 | SLC33A1 | FAIM | NECAB3 |
| NPC1 | MTM1 | GNL3 | SLC25A24 | SEC24C | TBL3 | RPL17 | PSMD7 | IKBKB | FGFR1 | SLC25A5 | SNORD102 |
| CISD2 | USP24 | ALDH2 | COLGALT1 | CUL3 | CMSS1 | EIF3L | ITIH4 | MIR30A | ARHGDIA | CSDE1 | MIA-RAB4B |
| NRBF2 | OXSR1 | CASP2 | PRPF6 | NR4A1 | CCDC50 | TPX2 | MYO1E | NLRX1 | MAP4K4 | EEF1D | ROMO1 |
| GSK3B | NSUN4 | GRB2 | SRSF11 | TLR9 | SF1 | BCYRN1 | SCAP | MIR216A | FAP | GCN1 | PDGFRB |
| TMEM59 | TRIR | HNRNPH2 | LYAR | MYH9 | ATP6V0E1 | KRT8 | KRT15 | DHRSX | HMGA2 | PRRC2C | PDGFB |
| MFN2 | MOGS | MYO1C | NKRF | HK2 | GABPB1-AS1 | ILK | CXCL5 | PSMB5 | ANXA6 | STX12 | NUP98 |
| TECPR1 | DNAJC9 | HSD17B12 | APOO | CAMKK2 | EBP | GLUD1 | YTHDF1 | PIK3R2 | GPS1 | MBNL1 | SLC22A4 |
| TRAF6 | ACADM | SNHG1 | NASP | MIR204 | BLOC1S1 | FBXW5 | MYL12B | SORL1 | MRTO4 | IFT20 | CRHR2 |
| LAMP1 | PCK2 | PPP3CB | TXLNA | DENND3 | PFKM | CETN1 | UACA | HDAC4 | ZFAND1 | IGF2BP3 | PNPLA8 |
| FUS | ZNF787 | HAX1 | CORO1B | ELAVL1 | PAX5 | MTHFD1L | HOXA11-AS | TRS-TGA2-1 | FGF1 | TBC1D7 | NCAPG |
| SOD1 | RPS6KA1 | SLC37A4 | ATP2A3 | RAB9A | SLC22A5 | KLHL20 | CTNS | CSNK2A1 | SERPINB2 | VPS33B | RNH1 |
| MEG3 | ECSIT | FASN | ATP6V1D | PHGDH | UBE2B | FLNB | MIR664A | FAS | SLC38A9 | CYC1 | LINC02489 |
| STING1 | EMC6 | DDOST | ARL8B | MIR30B | LYST | ASPH | MYOT | CYCS | CREB1 | IPO5 | LINC02490 |
| MLST8 | LNCRNA-ATB | APAF1 | APOLD1 | PLIN3 | NUCKS1 | RRM2 | PSENEN | PHF23 | LRRFIP2 | ATP6V1H | STK32A-AS1 |
| MAPK8 | MIR103A1 | NUAK2 | PSMD8 | IRF1 | SH3GLB2 | COL18A1 | AP1B1 | PICALM | SNRPA | PTK2 | SLC1A2 |
| UBQLN4 | MIR379 | CTSK | CD27-AS1 | JMY | TBC1D9B | MIRLET7E | RNF152 | HDAC9 | TSPAN1 | RPN2 | SORT1 |
| APP | TLN1 | MCM7 | MIR496 | EIF5 | MIR206 | RTN4 | PRKG1 | TMEM39A | MCM3AP-AS1 | TNIP1 | RBFOX3 |
| EMSLR | CEP170 | NDRG2 | OCRL | MIR211 | TRPC6 | SEC22B | MRE11 | MIR21 | PURA | EFTUD2 | CHCHD6 |
| TUFM | AMOT | ZDHHC12 | EPS8 | OFD1 | FGF7 | CFL1 | MECOM | ACBD5 | TBCK | RPL27 | PLIN1 |
| TOMM40 | MIR638 | PIP4K2A | SHARPIN | ACSS2 | PFKFB4 | MAPRE1 | AFP | ATF4 | KCTD7 | PCLAF | GCLC |
| SMCR8 | RPL31 | ITGB2 | PPP2R5D | CANX | MUC5AC | PSMA5 | DGAT1 | RAB1B | ANKHD1 | MIR20B | CERS1 |
| BAG3 | APEH | CAD | LIG3 | PRKAB2 | RMST | YTHDF2 | AKR1C2 | EEF1A1 | LINC02488 | NUP155 | FPGS |
| HIF1A | SMC2 | RPS15A | WDR11 | MAPK3 | MIR495 | RPL21 | PPP2R2A | SNX14 | MIR501 | PFKFB3 | CAP1 |
| CHMP2B | SMARCC2 | MIR16-1 | PDCL | UBE2L3 | RILP | RAB15 | SET | WDR47 | LINC-PINT | GMPS | IRGQ |
| RNF216 | EIF2B1 | SNORD44 | TMEM209 | CCT3 | MAP2K7 | IFI30 | CHKA | YWHAQ | JUNB | TFAM | ATAD3C |
| TECPR2 | LDLR | MYBBP1A | ERCC3 | RIGI | PABPN1 | AZGP1 | GJB5 | DAPK2 | MPDU1 | VPS26A | KBTBD7 |
| ZKSCAN3 | CDC42 | AHR | TPI1 | SEC16A | CPSF3 | MTMR6 | LAS1L | MAP1S | PNP | CDK5RAP3 | MCMBP |
| MAPK14 | STOM | CLRN1-AS1 | POLR2A | TRV-AAC1-4 | LMO7 | CCNB1 | DNAJC11 | G3BP1 | RALA | TPM2 | MIRLET7F1 |
| BCL2L1 | SLC1A3 | CREBBP | CYP51A1 | HSP90B1 | BTF3 | NDUFA13 | GTF3C3 | ENSG00000284820 | ATP6V0A2 | H2AX | STAMBP |
| CASP3 | ABCA1 | LBR | CLPX | WNT5A | ASF1A | KDM4A | MRPL37 | USP36 | AP1S3 | POLRMT | CDKN3 |
| MAPK1 | TTK | IGFBP3 | NDUFAF4 | VPS4A | TTI1 | KAT2B | ACTBL2 | HDAC2 | SBSN | LMAN1 | CLIC4 |
| APOE | SLC27A4 | BCL10 | SEL1L | LMNB1 | MRPL22 | AGO2 | GNL3L | FNIP1 | APOA1 | CD4 | LLGL2 |
| VAMP8 | MGST1 | XRCC6 | ARPC5 | MIR130A | CAAP1 | MTCL3 | DEFB103B | TAF15 | CD209 | WARS1 | PRAM1 |
| ULK3 | VANGL1 | TBC1D15 | DIS3 | NPM1 | G6PD | DAPL1 | B2M | TNFSF10 | RHOB | CUL1 | MPHOSPH8 |
| USP10 | KLHL9 | MIR215 | EIF3E | CHKB-CPT1B | PIAS3 | GABARAPL3 | MAP2K4 | SPTAN1 | GPC1 | PXN | RBPJ |
| GBA1 | SENP3 | TSPAN8 | FARSA | FNIP2 | CLTCL1 | RMRP | KIF11 | PLEKHM1 | VARS1 | RPL6 | EFNB1 |
| CTSD | H2AZ2 | SYNPO2 | ARIH1 | CPTP | ADI1 | TGFBRAP1 | SLC4A1 | CD44 | ALDH9A1 | STX7 | ERAP1 |
| MTMR14 | NLE1 | CHCHD3 | POLR1B | NBR2 | NUCB2 | PPP1R13L | CSNK1E | IRF3 | NUDC | U2AF1 | DARS2 |
| TSC2 | WDR43 | LYPLA1 | DIP2B | TFRC | CTU2 | DHX30 | SLC29A1 | CGAS | EIF1AX | NEK9 | PIGK |
| NLRP3 | LINC00336 | NR1H4 | CIR1 | RBX1 | GLTP | IL33 | FECH | VPS41 | PKMYT1 | MAP4 | PTDSS1 |
| GAS5 | TBC1D7-LOC100130357 | GLA | DDX52 | ITGB3 | DDX46 | USP5 | PTK6 | TUBA1B | IFNA1 | TOP2A | ADSS2 |
| TUBA4A | FAM13A-AS1 | DCAF12 | MRPS18B | RNF5 | PPP6R3 | SVIP | PTPRA | TRAF2 | WBP2 | MIR19A | EARS2 |
| GAA | RHOA | MIR16-2 | PDIA5 | PAWR | BORCS5 | C3 | NFS1 | PSMA3 | ZFR | SLC16A1 | RHOT1 |
| HDAC10 | PDGFA | RPLP2 | NOM1 | MIR181A1 | MIR488 | TRAPPC12 | POLR3A | SMAD4 | FAM98B | STMN1 | AKAP1 |
| PFN1 | COPS5 | RPL7A | NELFE | IKBKE | MIR508 | DST | UBE2A | S100A8 | EBNA1BP2 | CACYBP | PLD3 |
| PSEN1 | CDR1-AS | NRAS | KCTD10 | NCOA4 | WAC-AS1 | GYS1 | DOT1L | NEFH | FAM98A | ALDOA | MAZ |
| USP13 | TNNI3 | CUL4A | POGLUT2 | TUBA1A | CT55 | PSMA4 | MED12 | PRPH | TMEM160 | TRPM7 | WASF2 |
| TGFB1 | USP35 | MIR144 | RBFA | MIAT | PRKD2 | NLRP4 | SNRPN | UNC13A | NOS3 | MIR126 | POLDIP3 |
| PTEN | DNAJB11 | EPHB4 | SYF2 | SNHG14 | EIF4G3 | HSPB6 | AP2S1 | MIR133B | PRKCB | MIR125B2 | RNF40 |
| TBC1D5 | SLTM | SERPINE1 | H2AC21 | RETREG2 | TPCN1 | SRSF3 | BAMBI | RHEB | SNAI1 | LINC00520 | SSR1 |
| NOD1 | MIR1290 | RAB27A | RNU1-1 | AP2M1 | GAB1 | NDUFA10 | CTNNA2 | ATXN1 | MCCC2 | RAB11B | BASP1 |
| STK11 | ADRB2 | ADAM12 | CDH2 | MET | CUX1 | BRIX1 | GADD45A | CLTC | RING1 | MIR24-1 | CD109 |
| PARP1 | CAMK2D | EPHA7 | ALOX5 | KPNB1 | TAB1 | CAPN1 | GGH | CFLAR | IST1 | USP7 | YTHDF3 |
| RAB8A | FLII | DFFA | MSH6 | BANF1 | SNAPIN | EIF3G | PMP22 | NOTCH1 | DDA1 | SEPTIN9 | MCTS1 |
| RPS6KB1 | DNAJC3 | DIDO1 | ZEB2 | NPRL2 | ZFYVE16 | EIF5B | SBDS | STUB1 | TMEM164 | DICER1 | MRPL15 |
| NFKB1 | BAP1 | RAB25 | GLDC | UBAP1 | MOB4 | FTSJ3 | SF3B4 | WAC | MIR31HG | ITGA2 | UBFD1 |
| TIA1 | HERC5 | TICAM1 | DLAT | PIKFYVE | TRA2A | ESYT1 | AHSA1 | AKT1S1 | PSMB2 | ARG1 | GPATCH4 |
| EGFR | PHF5A | PLEKHF1 | BCS1L | EEF2 | PLCG1 | KIF5C | CLP1 | TRE-TTC3-1 | TRA2B | EEF1B2 | MRPL30 |
| TRIM21 | MIR192 | MIR199A2 | IDH3B | PLIN2 | ATP1B1 | HLA-DPB1 | MAN2A1 | HSPB1 | UFSP2 | MIR22 | PTRH1 |
| SPG11 | TTC5 | SEC23A | LGMN | MTMR9 | UQCRFS1 | HLA-DPA1 | S100A6 | ITPR2 | NUP210 | USP9X | AKAP17A |
| HNRNPA1 | KDM2B | GPR137B | UBA2 | SERPINF1 | MAP2 | HLA-DQA2 | TUSC3 | RAB23 | CCDC86 | SP1 | H4C14 |
| MCL1 | HSPBP1 | GPR137 | UBASH3B | MAPK9 | UBE2T | HLA-DQB2 | CYB5B | VRK1 | CXCL12 | ERGIC1 | MIR17HG |
| DEPTOR | HK1 | TUBB2A | DNAJB4 | VPS35 | PLSCR1 | RAB32 | LGR4 | SMAD3 | FLNC-AS1 | ATP5F1C | TMEM39B |
| KRAS | MLH1 | TOMM5 | WDR62 | TPCN2 | MIR19B1 | KPNA3 | AKR7A2 | CLN3 | MIR181D | GLS | C11orf98 |
| MATR3 | PGM1 | LINC01871 | IBTK | TBKBP1 | MIR374B | ADRM1 | PIGO | RACK1 | RAB11FIP1 | PDK1 | APLNR |
| TRIM27 | XDH | ADIPOQ | UXT | CD5L | MIR516A1 | DCST1-AS1 | PPIG | STK4 | DCAF17 | FTL | SNRPB |
| PVT1 | ADSL | PANX1 | TNKS1BP1 | MIR451A | MIR761 | LOC654780 | ACP2 | UBE3A | AGFG1 | LINC00470 | CDK11B |
| RAB5A | DSG1 | CTF1 | TBC1D16 | MIR543 | SNW1 | NUP50 | IQGAP2 | PIM3 | STX4 | RPA2 | CCL26 |
| TRC-GCA24-1 | H2BC21 | FBXO7 | IGHG2 | MIR140 | LOC116216141 | USP33 | LMAN2L | UCHL1 | RPS12 | DYNC1LI1 | TCF20 |
| VEGFA | HBS1L | PSMB9 | TBC1D19 | CCAT1 | LOC116216142 | MUL1 | MICA | S100A9 | DDX10 | DMXL2 | SMU1 |
| HSPB8 | WDR36 | HDAC11 | MIR9-2HG | MFSD8 | LOC116216143 | KLF4 | MPZL1 | PDCD6IP | CEBPZ | PCBP2 | OTUD7B |
| VAPB | WDR12 | EPM2A | MIR218-2 | MIR30D | LOC116216144 | MIR212 | PPP1R8 | LRSAM1 | RALY | PI4K2A | MIR590 |
| HULC | UBA5 | MOAP1 | SMIM31 | SIRT3 | LOC116216145 | MIR24-2 | CERS6 | CTNNB1 | TM9SF4 | SRSF7 | MIR365A |
| EVA1A | SYK | EEF1G | SEPT5-GP1BB | RAB35 | LOC116216146 | FLOT1 | LDB1 | WDR6 | SARNP | FIRRE | STAT5B |
| WDR45B | CHAF1A | NUP93 | COL5A1 | MIR224 | LOC116268446 | PDCD11 | OTUD5 | DYNLL1 | CCDC12 | NFKBIA | CAST |
| HNRNPA2B1 | NGF | TRAPPC5 | LAMA5 | LDHA | LOC116268447 | EIF4A1 | RBMS1 | FHL1 | UTP3 | TRPV1 | GLO1 |
| MCOLN1 | ALCAM | PCBP1 | TNKS2 | PSIP1 | SGTA | SERPINH1 | TOR1B | ERN1 | AGT | FHL2 | SUMO2 |
| ENDOG | HIPK3 | PWAR4 | GSTK1 | KHDRBS1 | NCLN | LAP3 | RPP30 | CHMP4B | WNT3A | SLX1A-SULT1A3 | CDIPT |
| LRPPRC | CHD6 | IL2RA | MORF4L1 | FKBP8 | UTP18 | TPD52 | EMC10 | E2F1 | SUCLA2 | CSTB | SEPHS1 |
| SIGMAR1 | MIR182 | MAPK10 | ARHGEF17 | MIRLET7C | AP4E1 | AARS1 | HSD17B8 | MAOA | GSTO1 | EHMT2 | MIA3 |
| IFNG | MIR384 | BIRC7 | UQCR10 | WDFY4 | HLA-B | MIB1 | MCRS1 | HRAS | PLOD2 | PPP1R12A | EHD4 |
| RAB1A | ABLIM1 | STK17B | APLN | TNPO1 | MPRIP | HM13 | VTA1 | RAB33A | TNIK | CAND1 | KCTD5 |
| ATP13A2 | SEC13 | LINC00324 | SNRPD3 | TUBB6 | ZCCHC17 | VPS37A | BLOC1S5 | DCN | NR2C2 | NAT10 | THOC3 |
| HTT | SMARCA5 | CHMP7 | ZNF106 | SKP1 | ZNF593 | MIRLET7G | LSM5 | TRIM13 | NUP133 | ZC3HAV1 | LYSET |
| RAB39B | PCNP | CHMP4C | RCN3 | PFKP | MAP3K1 | MIR27A | MTG2 | VDAC2 | S100A11 | ITGA6 | WAS |
| FOXK1 | PRDX6 | PAICS | CNPY2 | ANXA5 | UROD | MIR9-1 | P3H4 | BBC3 | STXBP3 | PSMA7 | CEBPA |
| PRKAB1 | CD46 | COPB2 | GMIP | RAB8B | XPO5 | FLOT2 | PWP2 | XIAP | U2AF2 | QARS1 | DDR1 |
| FOXO1 | FYN | TXNIP | MIR7-3 | SLC35D3 | EEF1E1 | NOP53 | RCL1 | TOMM20 | STK25 | CD84 | PLD2 |
| XIST | GFER | GANAB | MIR7-2 | AR | LSM2 | DAB2 | UTP6 | EP300 | NLRC5 | RPS14 | ROCK2 |
| TRIM5 | CD82 | PRMT5 | IGKV2D-29 | XPO1 | RPS21 | IFI27 | GOLIM4 | RAB12 | CRNKL1 | GTPBP4 | EIF4A2 |
| DNMT1 | KRT19 | MT2A | SERF2-C15ORF63 | COL1A1 | PTPN12 | SMC1A | DDX49 | ATF6 | ANP32E | TJP1 | SCO1 |
| BAX | MDH1 | UFL1 | DDRGK1 | PSMC4 | ASNS | RANBP1 | HERC4 | PRKAG2 | MIR542 | SNRPD1 | CNP |
| CASP8 | CUL5 | PSMC3 | MIR369 | LGALS1 | ABCB7 | MLLT11 | PPWD1 | RB1 | HCG18 | ATL3 | IL18 |
| IL6 | MRPL3 | RPS17 | TF | RAN | LTA4H | PI4KA | TMED2 | SH3BP4 | MIR1291 | TRAPPC11 | FANCI |
| TLR4 | AGR2 | TLR2 | AVL9 | SGPP1 | RCAN1 | EGOT | TTC1 | PRKDC | TRMT112 | NHLRC1 | SYNE2 |
| SIRT2 | ASCC3 | TBC1D25 | MYH11 | EIF4B | KRT9 | PDHA1 | XXYLT1 | HSPA4 | BACE1 | RTN3 | EIF2S2 |
| ATM | BMS1 | PSAP | PRMT6 | TM9SF1 | PRPF31 | TAGLN2 | FNDC3A | ATP6V1A | SMPD1 | TRIM6 | RHOT2 |
| DAP | MISP | RTCB | OTULIN | CTSL | GSPT1 | GDI1 | MAK16 | WASHC1 | AIMP1 | NUMB | TAOK3 |
| FLCN | LOC126806603 | DSC1 | TELO2 | DNAJA3 | RBP1 | RNF31 | NPM3 | CEBPB | MEF2D | PSMD13 | MYO1D |
| ATXN3 | PIK3R3 | SFTA3 | SNRPF | MAVS | STIM2 | PPP1CB | PARD6B | TUBB4B | SDHD | LGALS9 | GOLGA3 |
| NUPR1 | CLN6 | PDIA6 | FDFT1 | PIK3C2A | WWP1 | RAD51 | RRP9 | LINC-ROR | H1-4 | NUP107 | MRPS34 |
| PIM2 | AAAS | LINC00963 | CD9 | IL10 | BCCIP | IRS1 | TMED4 | VPS16 | RPL35 | ZNF418 | RIC8A |
| DAPK3 | OGDH | ZMPSTE24 | TFDP1 | CDK1 | IPMK | DBN1 | CCDC93 | TSG101 | S100A10 | UQCRC1 | AFG2B |
| HDAC1 | DKC1 | TERT | CDC27 | OLR1 | BZW1 | WDR83 | CEP170B | PANK2 | MACF1 | RPS11 | ERLEC1 |
| MAP1LC3B2 | HCFC1 | TSPO | ERO1A | UBR4 | TRMT61B | MROCKI | CHTOP | TMEM150B | RPL35A | SMURF1 | PDXDC1 |
| SETX | TRRAP | MIR183 | SAR1B | ANXA1 | MIR526B | CCZ1 | H2BC11 | PIK3CG | CCN1 | TRIM8 | RABL3 |
| TRAPPC4 | PARN | LINC00665 | TGM3 | DLEU2 | SF3B3 | PRKACA | TMED7 | MYO6 | MRTFA | MIR22HG | SEPTIN11 |
| DCTN1 | AIMP2 | SPTLC1 | FBXO32 | PPP2CA | SNRPB2 | IRF5 | H3C14 | HOTAIRM1 | MVP | DDX39A | RPL22L1 |
| TRIM65 | MRPS23 | SNAP23 | RAB37 | CASP14 | HP1BP3 | CLCN3 | IGKC | OPA1 | SERPINB3 | RCC2-AS1 | TRIM55 |
| SRC | RPL37 | NFATC1 | NOL6 | CCT8 | ATP7A | HUWE1 | RNF149 | UBA52 | ATPAF1 | MYH10 | GPX3 |
| GFAP | SPCS2 | GZMB | FBXO2 | TRP-AGG2-5 | LNPEP | CASR | WASHC2C | PON1 | RTRAF | MTMR4 | SUB1 |
| HSPA5 | TRIM49 | FERMT2 | PAK1IP1 | ISG15 | GAN | CCL2 | ENDOD1 | KIF5A | LAPTM4B | RPN1 | MIR135A1 |
| CTSB | UGDH | CDIP1 | STK11IP | HOTAIR | RPL30 | RPL5 | METTL17 | GLE1 | LARP4 | MIR214 | SNHG15 |
| TRIM32 | MIR92B | GNB2 | NOL10 | IL6ST | NGFR | SLC25A1 | NIPSNAP3A | TANK | NGDN | PDIA3 | MIR423 |
| PRNP | IFIH1 | RPA1 | NOP16 | EEF2K | LRP6 | ERLIN1 | MIR382 | CXCL8 | PGGT1B | BTRC | FALEC |
| MYC | USP32 | TAB3 | RRP36 | VPS29 | SLC2A4 | HNRNPL | MIR551B | RAB18 | KLHDC10 | NOP58 | MMP1 |
| CDKN2A | MIR301A | CD40LG | UTP20 | PPP1CA | CCL5 | OAT | HSP90AB4P | YWHAB | NRBP2 | DLEU1 | BAZ1B |
| AURKA | MIR153-2 | RAB41 | NCCRP1 | SNHG16 | TREM1 | NRP1 | KCNMB2-AS1 | PIK3R1 | M6PR | DSG2 | TXNDC17 |
| TNF | RAB29 | STRIP1 | DOCK1 | IFT88 | TTF2 | FBL | MIR3187 | CRY1 | KRT2 | DDX54 | NOL11 |
| IL1B | UBE2L6 | SLC2A1 | GNS | COPB1 | ERAL1 | MANF | LINC01093 | GAPDH | CALML5 | PCNT | DEDD |
| PIK3CA | MIR185 | PRKCD | KRT4 | NDRG1 | CEP131 | RPL9 | MIR3185 | CASP7 | IRF8 | HLA-DQA1 | MIR760 |
| VPS39 | PPP2R2B | CCT5 | CYLD-AS1 | MIR199A1 | OSGIN1 | LEPR | DMD | JUN | COL6A1 | SEC62 | NTHL1 |
| ALS2 | HNRNPDL | SND1 | NDUFB9 | PGK1 | MIR10B | SNX7 | ELANE | CBS | HAGLR | MIF | SNCG |
| HNRNPD | RNF115 | TUBB2B | CRBN | ITGA3 | DLX6-AS1 | RUFY4 | STAT2 | HFE | FGD5-AS1 | UTRN | MYO18A |
| TMEM208 | TPT1 | PROM1 | FABP6 | DAXX | DRAIC | SRPK1 | C5 | STIM1 | EIF3J-DT | CDK6 | TXNL1 |
| TBC1D14 | RIF1 | SLC25A6 | SH3GL2 | RAB2A | NUP214 | MIR361 | GCDH | RNF185 | IRF7 | SEC23B | MYO10 |
| HMOX1 | RRP1B | RPL7 | MYL12A | TRAPPC8 | VAMP2 | TP53BP1 | NME2 | MIR34A | MAX | SEC24B | PPIL4 |
| VIM | MIR149 | CYTOR | STRIP2 | MYCBP2 | NRDC | GLT8D1 | NPR3 | BIRC6 | MYCN | PES1 | MYO5C |
| DNM2 | ABCF1 | HACD3 | FUBP3 | BID | RPL24 | PDIA4 | PLA2G1B | UBB | IL1A | AP1M1 | MIR208B |
| PSMD4 | RPS27L | LRRC59 | PALS2 | FLNC | PPAN | MPP2 | UBE2K | VPS11 | VTN | IDH2 | LIX1L |
| LGALS8 | SORBS3 | HGF | BORCS7 | TRIM11 | CFAP20 | EIF4E | PRKAR2A | RNF166 | ARHGAP4 | RRP12 | VTI1A |
| FIG4 | PRRC2B | CXCL1 | HMBOX1 | RAB38 | MRPL4 | CYLD | MUC2 | MIR20A | DUSP4 | SAFB | NPRL3 |
| GRN | MIR199B | IGFBP5 | BORCS8 | PDPK1 | FCF1 | ACTC1 | SCRIB | MIR17 | ZFP91 | RPL18A | BLM |
| EWSR1 | TRV-CAC1-2 | COL10A1 | ISOC2 | MYD88 | GUSB | EMC1 | SUPT5H | RETREG3 | SPOUT1 | TKT | DHFR |
| ASAH1 | LIMA1 | TNFRSF12A | TTN-AS1 | RAB13 | MAGED1 | IRAK1 | CPNE1 | YWHAG | CALM2 | DARS1 | KRT1 |
| BNIP3L | DERL1 | TNFSF12 | VIM2P | TLK2 | MELK | PRODH | ERAP2 | PSMB4 | POLR1A | GART | CBFB |
| LINC02605 | RUSC1 | URI1 | CCL18 | RASIP1 | FOXP1 | KIF23 | REEP2 | CDKN1B | TAF6 | H1-2 | CARD9 |
| RNASEL | NF1 | VAMP7 | MIR100HG | PSMD2 | WASF1 | RASSF1 | SIRT4 | EPAS1 | RP9 | CD47 | HMOX2 |
| MALAT1 | CSTA | CHMP4A | DDX11-AS1 | RAB26 | NIPBL | PEX13 | PLA2R1 | ITM2B | FKBP15 | MAT2A | CELF2 |
| TUG1 | HARS1 | SIRT5 | CLN8 | CAPNS1 | RPIA | DCAF13 | TMX3 | ITGB1 | PNPO | GBF1 | EFEMP1 |
| GOPC | CLN5 | EIF3F | LMCD1 | IFNL2 | MIR4284 | RAD23B | MIR330 | TMEM199 | KDM2A | STK38L | NSUN2 |
| GSN | DHX16 | EIF2A | SESN3 | CCT4 | PRRT3-AS1 | FKBP5 | MIR154 | MAP1B | TRAPPC2L | LRP1 | PISD |
| GNE | EPB41L2 | MPG | ERVW-1 | ZC3H12A | SPAG5-AS1 | NDUFS1 | HSPA7 | INSR | H4C1 | ACTG1 | SERPINB6 |
| HSPA9 | GLIPR2 | RASSF5 | TCHP | DEPDC5 | MIR5100 | ALDH18A1 | RN7SL1 | RRAGB | UBL4A | ACTR2 | NAXE |
| NLRP6 | TBC1D10A | SNHG12 | HECTD1 | KPNA2 | MIR581 | MBP | LINC01139 | RAF1 | RCC2 | PKD2 | UBR5 |
| TMBIM6 | LINC00641 | MIR124-1 | GAS8-AS1 | PTRH2 | LOC130056519 | SLC25A10 | RNU4-2 | FADD | GAK | MIR26A1 | PSMD9 |
| KIF25 | TTTY15 | RAB3GAP2 | PRECSIT | BCL2L10 | DSC3 | RPS8 | WSPAR | SIRT6 | AKR1C3 | TP63 | CBX5 |
| WWOX | HES1 | ETFA | SNAP25 | MIR210 | MCM4 | PGRMC2 | LINC01806 | PMAIP1 | MIR141 | RPS24 | OS9 |
| XRCC5 | EXOC2 | MIR99B | VKORC1 | CREG1 | ALDH3A2 | CPT1A | MIR6852 | LINC00941 | MIR124-3 | RPL23 | ETF1 |
| TRIM22 | ARF3 | LINC01063 | CCNH | BMPR2 | DOCK2 | SOD2 | DPM1 | STX5 | PRKCA | MIR186 | RCN1 |
| GOLGA2 | ERGIC3 | NUFIP2 | ANK1 | LMNB2 | KRIT1 | PSMD14 | ARNT | ITGB4 | PRCP | SGPL1 | STX2 |
| RASA2 | TRAPPC13 | ACTN1 | ARID1A | TMED10 | MLEC | PAQR3 | ARSA | MON1A | TOMM6 | SRP54 | EMG1 |
| MIR106A | YOD1 | TAPBP | SRF | RAB4B-EGLN2 | MED10 | TFAP2A | ARSB | RBM8A | PEDS1-UBE2V1 | LUZP1 | YTHDC1 |
| ATP6V1B2 | CCND3 | FAT1 | LMX1B | LDHB | MED6 | IL7R | C12orf44 | RNF168 | LINC00467 | ADAR | CAMK2G |
| SPATA33 | ARRB1 | APOL1 | MED23 | SH3BGRL | MED7 | MAGED2 | C17orf88 | MTPAP | SRM | AGL | ASIC1 |
| UPF1 | VTCN1 | EXOC8 | RNMT | MIR23A | RPL39 | RAD23A | CAPN10 | RPS25 | MT-CO2 | NUMA1 | CD24 |
| TRAF3 | FAM131B | RRBP1 | ALOX12B | GRAMD1A | MED8 | PCAT1 | CTSL1 | TGFBR2 | STT3B | EEA1 | MIR497 |
| SLC3A2 | FBXO21 | FXR1 | CTDP1 | CHEK2 | POLR1F | SSR4 | CX3CL1 | CALU | KIDINS220 | HNRNPH3 | ARHGAP27P1 |
| TTR | LINC01619 | INHBA | P2RX6 | MIR145 | SDAD1 | NEFL | DLC1 | RHOG | DLST | PIAS4 | TUBB1 |
| SCFD1 | MIR26A2 | TNFRSF11A | PIGR | CASP4 | GOT2 | CD36 | DNAJB9 | DNAJA2 | SHMT2 | RPL12 | RNASE1 |
| IL17A | MIR511 | BMP2 | UROS | ACTB | APRT | IPO7 | ERO1L | RPS18 | PRDX4 | TERC | TUBA3E |
| MIR143 | MPDU1-AS1 | AGPS | VARS2 | HOTTIP | MMAB | STK38 | FAM48A | NCBP1 | SEPTIN7 | MIR654 | TOMM7 |
| TOMM22 | LINC03088 | RPL19 | BRF1 | IARS1 | NEK7 | MIR137 | FKBP1A | USP19 | CLPTM1L | CHMP1A | TUBA3C |
| MLKL | CYP2E1 | HEATR1 | NOP10 | SNHG5 | ARPC1A | SGK1 | FKBP1B | MIR188 | U2SURP | KIT | TUBA3D |
| FMR1 | KARS1 | SFXN1 | ARL2 | PPARA | TRMT1L | TRAPPC3 | FOS | MIR506 | SMARCA4 | IGFBP7 | ATP5PD |
| ITGA5 | NDUFS7 | WDR3 | CCDC22 | MIR101-1 | MIR133A1HG | HYOU1 | KIAA0226 | AFG3L2 | PFAS | IL12RB1 | SLC7A11 |
| LAMP3 | PLOD1 | SOX2-OT | FOXE1 | TP53BP2 | OCLN | RALBP1 | KIAA0652 | SPNS1 | RPL36 | IL5RA | STIP1 |
| NOP56 | ARPC2 | SREBF1 | MPST | PSMB8 | ECM1 | HMGN2 | KIAA0831 | NSDHL | HNRNPA0 | ITGA1 | HERPUD1 |
| PPP1R15A | PUF60 | IGF2BP2 | POLR2L | HDAC5 | PEG10 | ALDH1A1 | KLHL24 | ENSG00000243902 | PHLPP2 | ITGA7 | ATP6AP1 |
| MIR146B | DHX36 | ALDH7A1 | UBA7 | H2BC3 | TIMM50 | PNPLA2 | MAPK8IP1 | CAVIN1 | SNX27 | PTH | RAB3GAP1 |
| RNF186 | PRPF4 | UBE2I | FUBP1 | ROCK1 | ARHGEF2 | RPL22 | MBTPS2 | CERKL | RARA | SEMA3A | ITPR3 |
| DYNC1LI2 | SPECC1L | TFG | GTF2B | ATP6V1C1 | NISCH | TFCP2 | NAF1 | EIF3M | TOM1L2 | SEMA4D | HNRNPUL1 |
| TCP1 | SHC1 | HMGB2 | KMT2B | KDM4B | PNKD | MIRLET7I | NCKAP1 | WDR18 | PSME2 | SMAD9 | NONO |
| SMAD1 | MCAM | STAM | PRSS21 | LAMTOR2 | POLD2 | BAG5 | NLRC4 | BUD23 | TBC1D2B | WNT10B | MIR455 |
| EIF2AK2 | ALG1 | TNFRSF1B | RFK | CSF1 | NOP2 | MIR29C | NRG1 | C1orf35 | DCTPP1 | CCR1 | SLCO4A1-AS1 |
| HNRNPU | BLMH | UNC5B | SUPT4H1 | AVEN | WTAP | MIR153-1 | NRG2 | MIR342 | MIRLET7A1 | TFAP2B | CLPP |
| STK3 | BAG6 | SHH | BRF2 | MIR125B1 | RBM26 | RFC2 | NRG3 | RBM39 | MIR378A | CALCA | ATAD3B |
| LAMTOR3 | ANKZF1 | MKI67 | GRPEL1 | HCCAT5 | PRF1 | PA2G4 | PARK2 | TPR | FSCN1 | DKK1 | ABHD5 |
| CCT2 | SNX6 | MYOC | PITPNB | CLOCK | SMN1 | PEDS1 | PRKCQ | FKRP | MIR193B | FZD9 | NAA10 |
| IL6R | AP4M1 | STX6 | RABGGTA | MAP2K3 | TPM3 | TUBB8 | TMEM49 | TBC1D10B | MIR582 | IL11RA | SLC1A5 |
| RNF41 | APBB1 | MTA2 | MARCHF5 | IL24 | PEX14 | MIR663B | TUSC1 | NSFL1C | LINC00987 | IL3RA | RPL34 |
| UBE3C | APC | SAFB2 | MED14 | RNF213 | P4HA1 | RPL10 | WDR45L | MID2 | POMGNT2 | ITGA8 | TBL2 |
| MIR494 | KAT2A | SEC31A | FBXO28 | HSPD1 | H1-0 | SMC4 | HCRT | ERFE | UCP2 | ITGA9 | ILF2 |
| HNRNPA3 | ATP7B | IL23A | GCA | CLU | COPE | IGF2BP1 | MARCKS | SNHG17 | PSMD6 | MIR7-1 | DIABLO |
| PHB1 | PTPN13 | NUDT21 | HERC3 | SBF1 | NHERF2 | PIGT | NOC4L | MIRLET7BHG | RBBP7 | STX8 | FAF1 |
| CKAP4 | LZTR1 | TNFAIP8 | MYLIP | DDX5 | AKAP8L | CSTF1 | PRKCSH | CDC14A | AP2A2 | DYRK1B | MAP3K5 |
| FLNA | NHP2 | RPL28 | QRICH1 | VTRNA1-1 | H3-7 | VPS37C | PBK | TRAPPC2 | NCSTN | VPS28 | COPZ1 |
| GBP1 | STRN | STYK1 | TRIM31 | DDB1 | WT1 | DSP | MACROH2A1 | RBM14 | ADAM9 | MVB12A | SNORD118 |
| SETD2 | UFC1 | ZNNT1 | KANSL3 | EZH2 | NPR1 | PC | RABGAP1L | PLEC | NSMAF | VPS37B | SUMO1 |
| HNRNPM | ATP13A1 | RAB17 | LSM7 | RPS3 | PADI4 | USP15 | FBLN5 | LUC7L2 | GNAS | SCARB2 | NAMPT |
| PLK1 | MDN1 | LAMTOR5 | MED28 | RAB6A | SNAI2 | SPI1 | MCM2 | AQP3 | ITCH | HSPH1 | LARS1 |
| TGM2 | CSTF3 | MIR30C1 | NIP7 | PIR | COPS3 | MIR216B | EZR | MCCC1 | RANBP2 | HGH1 | SPARC |
| CASC2 | PLK3 | HPRT1 | RSL24D1 | PRKCI | CAPZA1 | XRN1 | HNRNPAB | SUCLG2 | EIF3B | APEX1 | EPHB3 |
| PI4KB | MYLK | DHCR7 | XPO6 | SNRNP70 | CLDN6 | NCBP2 | XAB2 | CDKN2B-AS1 | MED4 | AATF | BCL2L2 |
| SNHG6 | POLR1C | ACP1 | RALGAPB | EIF4A3 | SOCS5 | RPL29 | PEG3 | TNFRSF10D | WHAMM | PTPN1 | METTL3 |
| MFF | IFITM1 | REST | RLF | CCT7 | PHLDA1 | HLA-C | TRIO | HTATIP2 | SRPRB | CHERP | ESR2 |
| YWHAH | SUMF2 | RPS26 | TAF1B | PLIN4 | ENTREP3 | AXIN1 | CDK4 | RAB31 | ITGAL | DHX37 | TAB2 |
| MIR375 | FIZ1 | ERP44 | ACAD11 | LRRC25 | MIR3120 | DPYSL2 | CPD | VPS37D | TNFRSF10A | FGFR2 | COMT |
| HNRNPH1 | SYT1 | PDHB | PLP2 | PTBP1 | IDH1 | FEZ1 | LYN | USP8 | TNFRSF21 | PSMD10 | MIR23B |
| SHOC2 | NR1H3 | RPA3 | TAF7 | SCOC | NACA | ITGAM | PPP1CC | SEC61B | IL13RA1 | RAB3D | RMC1 |
| NUP62 | HPSE | RPLP1 | ZNHIT1 | MYL6 | DEF8 | PER1 | SOAT1 | SAR1A | MDH2 | DDX55 | FXR2 |
| HSPA1A | CYB5A | SYNCRIP | CNN2 | CCT6A | TMED9 | BHLHE41 | DDX20 | STOML2 | ITGB5 | DUSP16 | SDCBP |
| MTMR8 | MAP3K14 | CLTA | LPAR5 | STAT1 | TP53TG1 | XAF1 | XRN2 | RARS1 | ITGB7 | ERCC6 | TET1 |
| SNX30 | GRP | BCLAF1 | MRPL33 | NEFM | LINC02901 | PPP1R9A | EIF3D | VPS51 | SP7 | MPP1 | GGCT |
| RAB14 | PHLPP1 | GEMIN4 | MED11 | ARMC3 | MIR1260A | PPT1 | LTV1 | AKT2 | BMP3 | FAM111A | ATR |
| MMP14 | PKP1 | GSDMD | MED18 | SMAD5 | LINC02257 | ATP5PO | MIR155HG | CTSA | CASP5 | F3 | SRSF1 |
| NUP205 | KPNA6 | RPS13 | ZNF160 | BMAL1 | LINC00578 | FLG | HCCS | HSPA1B | FZD10 | WDR48 | USP11 |
| TRAP1 | PMEPA1 | DDX21 | ATXN7L3 | SCD | LINC00992 | FBXO22 | CCDC88A | UBA1 | GZMA | TWF1 | CASP6 |
| MAGEA6 | ZC3H15 | NKX2-3 | EIF1AD | SLC25A3 | LDB3 | CNOT1 | ATIC | PPARG | IL21R | LSM6 | TNFSF15 |
| ATP6V1G1 | RBM34 | ROS1 | FIBCD1 | RAB10 | PLS1 | GEMIN5 | PDCD5 | FURIN | SOST | TDRD3 | FZD2 |
| ATP1A1 | MIR545 | MIR659 | LSM8 | EIF4G1 | PIP | ILVBL | HMGN5 | RUNX2 | TNFRSF25 | IMP3 | ITGAX |
| SNX4 | ATF3 | SDHA | POP7 | VAPA | KRT83 | AKT3 | HSPA2 | HDAC3 | BMP5 | NUDT16L1 | TYMP |
| MIR373 | SLC1A4 | YARS1 | RSRC2 | CHEK1 | EIF1AY | GOSR1 | EIF2B3 | DDX3X | CCL3 | PSAT1 | CCR2 |
| SYT11 | HCRTR1 | SLC25A11 | ARMC6 | RMDN3 | ALB | TPP1 | POLR2B | MIR26B | CIB1 | SLAMF1 | COX4I1 |
| CSNK1A1 | CLDN5 | SEC11A | CENATAC | MIR502 | SRPK2 | GPSM1 | PCMT1 | ITGAV | VPS13A | COL8A2 | FZD1 |
| PCNA | C1QTNF9 | RPF2 | COMMD3 | MIR18A | METAP2 | PCYT1A | RAI14 | FOXP3 | OIP5-AS1 | SRMS | FZD5 |
| BHLHE40 | HCG27 | TGFB2-OT1 | MRPL17 | RICTOR | MYL9 | CCNA2 | CERS2 | NEU1 | UBE2D1 | DDIT4L | MIR205 |
| BMF | MIR29B2 | SPOP | NBPF15 | ING1 | CLIC1 | SUGT1 | TUBG1 | B4GALT1 | ARFIP2 | GPRASP1 | PRKAG3 |
| SFPQ | LEF1-AS1 | TRIM25 | RAVER1 | NR1D2 | TRIP12 | MIR33A | WDR26 | SMS | SERPINA1 | TINCR | SIK2 |
| ANXA7 | RNY3 | ATXN2L | RPP14 | MARS1 | TAF2 | CKM | RACGAP1 | TAOK1 | CUL4B | PCED1B-AS1 | VPS13D |
| PEA15 | CFTR-AS1 | POP1 | UBE3D | USP22 | UFM1 | DDX47 | TPM4 | RPS20 | RPL4 | TMPO-AS1 | TRPM2 |
| FBXW7 | MIR1298 | CCAR1 | COMMD2 | RPS4X | DBNL | EIF6 | MIR106B | USP1 | PAK1 | MIR103A2 | TRIM50 |
| CHUK | MIR513B | IMPA1 | RPP21 | ACE2 | SF3A1 | GSTP1 | CBX3 | THOP1 | MIR15A | PLAU | NAPA |
| BECN1P2 | ARAF | VRK2 | SYS1 | FAF2 | SF3A2 | AHNAK | LMAN2 | COG1 | P4HB | CDC25B | PFN2 |
| ACIN1 | CDK9 | FAU | COMMD3-BMI1 | MIR98 | AKAP11 | ELF1 | EXOC4 | GAS8 | THBS1 | RAB2B | HRNR |
| PWAR1 | EIF2B2 | MRPS2 | DNLZ | BMP4 | RTF2 | CHMP5 | MAP3K11 | NFE2L1 | FOXM1 | PLAA | FLT1 |
| TLR3 | MED17 | MTERF3 | MIX23 | SPHK1 | PRDM1 | TOR1A | PRMT1 | WWTR1 | SPP1 | RBM28 | PSMB3 |
| BMI1 | GATAD2A | KDR | MIR132 | CHMP3 | HSPA13 | CARS1 | ADNP | BABAM2 | CD274 | MIR100 | ATP2A1-AS1 |
| LINC01554 | MAP7 | PLD1 | MIR194-2 | TOMM70 | IL2RB | EDEM1 | DNAJC13 | MCUR1 | ATP6V0C | AKR1B10 | STARD7-AS1 |
| ANXA2 | RAB11FIP5 | CLDN1 | NR1D1 | COPA | ITGA2B | STC2 | MLF2 | PNN | GJA1 | MARCHF8 | MIR365B |
| USP30 | EXOSC1 | MYOF | PCLO | YBX1 | EPHA4 | SF3B1 | NAGLU | SAV1 | PSMC1 | MIR483 | LAMTOR4 |
| EPHA3 | PDCD6 | RPS10 | TMEM97 | RAB4A | FZD4 | RBM25 | ABCF2 | NRSN2 | RNF2 | OTUD6B-AS1 | PRKAR1A |
| SIDT2 | ESYT2 | ALYREF | ANKRD49 | IPO8 | ITGA4 | CDH1 | RPL36A-HNRNPH2 | TM9SF3 | MIR148A | SOS1-IT1 | RPL10A |
| TUBA8 | VPS13C | HNF1A-AS1 | PJVK | TPM1 | LRP5 | NTRK1 | JPX | PRPF38B | GNB1 | FBXL4 | KHSRP |
| NEAT1 | IKBIP | CALM1 | SIDT1 | MIR33B | PLAT | CAPN2 | SEC24D | AKAP13 | ACTL6A | SCAMP3 | DHX15 |
| TUBB | LSG1 | HLA-DRB4 | WASH2P | SMAD2 | ITGB6 | SMARCB1 | TERF2 | AP3D1 | COPG1 | IMP4 | SRRM2 |
| CPT2 | PCID2 | CLDN2 | WASH3P | DNAJC7 | EPHB1 | EIF3C | PLS3 | PPFIBP1 | ATP1B3 | CCZ1B | THRAP3 |
| RAC1 | EPPK1 | MIR449A | ENSG00000254692 | WDR24 | IL4R | GATA4 | GOLM1 | SURF4 | CLPTM1 | BRI3BP | KTN1 |
| ARG2 | HSBP1 | MIR498 | ENSG00000259522 | BST2 | CSF3R | KDELR1 | NOL9 | SNX17 | LOC126862737 | SESN1 | MIR671 |
| MALT1 | MAP1A | CDK5 | RAB24 | S100A4 | MEG8 | DYNC1I1 | LINC00313 | ABHD10 |  |  |  |

**Table S3.46 upregulated differentially expressed genes**

| ZNF587 | PTMS | LOC100190986 | HOXA6 | GOLGA8A | SPN | FBXW4P1 | LOC100129917 | BMI1 | FIZ1 | COL7A1 | ZNF835 |
| --- | --- | --- | --- | --- | --- | --- | --- | --- | --- | --- | --- |
| HIATL2 | FLJ16779 | HOXB4 | WFIKKN2 | ASPHD1 | GNL1 | MUC5B | FEZF2 | PTCHD2 | SNORA37 | SNORA71A | CCDC125 |
| EEF1D | RXRB | MUC2 | TAPBP | IRF7 | BRD2 | SNORD123 | ZBED1 | PBX2 | TIMM22 | MDC1 | DDR1 |
| MDFI | KCNK6 | ESPNL | ACTN3 | PTCH2 | MYBPC2 | TMEM179 | GABBR1 | ZAN | RPS18 |  |  |

**55 downregulated differentially expressed genes**

| MALAT1 | RTKN2 | CPEB3 | ANGPTL3 | OCEL1 | RNF103 | RAPGEF4 | CDH9 | FBXO21 | FAM174A | ROR1 |
| --- | --- | --- | --- | --- | --- | --- | --- | --- | --- | --- |
| KLF15 | PTPRD | SLC22A4 | CHCHD4 | CDH2 | ATP5D | MUC13 | MYCT1 | C3orf18 | LOC285768 | GSTA1 |
| GRIA3 | EPHX1 | FOLH1 | F11 | GATM | ETNK2 | CLSTN2 | CTXN3 | CYP17A1 | F2R | GC |
| SLC16A1 | IL1RL1 | GOT1 | KMO | HRSP12 | KCNH6 | CISD1 | DAO | AFM | PCOLCE2 | CPNE8 |
| GLRX | FOLR1 | DPEP1 | ALB | FAM151A | FGB | PLG | LYPD5 | RALYL | TRPM6 | SLC13A2 |

**Table S4.17 ARGs-DEGs**

| MALAT1 | BMI1 | FIZ1 | FBXO21 | SLC22A4 | CDH2 | SLC16A1 | CISD1 | DAO | EEF1D | MUC2 | TAPBP |
| --- | --- | --- | --- | --- | --- | --- | --- | --- | --- | --- | --- |
| IRF7 | ALB | MDC1 | DDR1 | RPS18 |  |  |  |  |  |  |  |
